# Method and its Composition for encapsulation, stabilization, and delivery of siRNA in Anionic polymeric nanoplex: An *In vitro*- *In vivo* Assessment

**DOI:** 10.1038/s41598-019-52390-4

**Published:** 2019-11-05

**Authors:** Nidhi Raval, Hardi Jogi, Piyush Gondaliya, Kiran Kalia, Rakesh K. Tekade

**Affiliations:** National Institute of Pharmaceutical Education and Research (NIPER) – Ahmedabad, Palaj, (An Institute of National Importance), Opposite Air Force Station, Gandhinagar, 382355 Gujarat India

**Keywords:** Pharmaceutics, Diabetic nephropathy

## Abstract

Small interfering RNA (siRNA) are synthetic RNA duplex designed to specifically knockdown the abnormal gene to treat a disease at cellular and molecular levels. In spite of their high potency, specificity, and therapeutic potential, the full-fledged utility of siRNA is predominantly limited to *in vitro* set-up. Till date, Onpattro is the only USFDA approved siRNA therapeutics available in the clinic. The lack of a reliable *in vivo* siRNA delivery carrier remains a foremost obstacle towards the clinical translation of siRNA therapeutics. To address the obstacles associated with siRNA delivery, we tested a dendrimer-templated polymeric approach involving a USFDA approved carrier (albumin) for *in vitro* as well as *in vivo* delivery of siRNA. The developed approach is simple in application, enhances the serum stability, avoids *in vivo* RNase-degradation and mediates cytosolic delivery of siRNA following the endosomal escape process. The successful *in vitro* and *in vivo* delivery of siRNA, as well as targeted gene knockdown potential, was demonstrated by HDAC4 inhibition in *vitro* diabetic nephropathy (DN) podocyte model as well as in *vivo* DN C57BL/6 mice model. The developed approach has been tested using HDAC4 siRNA as a model therapeutics, while the application can also be extended to other gene therapeutics including micro RNA (miRNA), plasmids oligonucleotides, etc.

## Introduction

Ribonucleic acid interference (RNAi) refers to a post-transcriptional gene silencing tool to neutralize or silence the pathological protein via activating RNA-induced silencing complex (RISC), endogenously. Gene allows clinicians to treat a disease by administering RNAi therapeutics (such as small interfering RNA (siRNA) or micro RNA (miRNA) into a patient’s instead of using drugs or surgical interventions^1^. However, the degradation of administered siRNA by circulatory RNase, their short half-life (t_1/2_) as well as rapid renal clearance are some of the prime challenges that complicate the clinical translation of siRNA therapeutics^2,3^. Further, the endo-lysosomal trapping of delivered siRNA is yet another key issue that results in the enzymatic-degradation of delivered siRNA leading to null-effect. Ideally, the siRNA delivery vector must find their way to escape from the endosomes/lysosomes compartment after entering inside the target cell to efficiently release the loaded siRNA in the cytosolic compartment^4^. Failure to overcome this barrier would significantly weaken or even totally eliminate the therapeutic effect of siRNA.

Initially, the viral vectors have been employed to deliver siRNA into the cell. However, the stimulation of the immune system through the activation of viral pathogens was found to be the foremost hurdle^5–7^. Tremendous efforts were made to develop a non-viral and clinically translatable approach for the intracellular transfection of siRNA^8^. In this context, Lipofectamine is the most widely employed siRNA transfection materials, but its application is primarily limited to *in vitro* set-up^9^. Some modified versions of lipofectamine have been developed, however, none of the existing modalities represents an ideal carrier to facilitate clinical translation of siRNA therapeutics. Invivofectamine and *in vivo*-jet-PEI are some of the potential siRNA carriers, but the applications of these agents are primarily restricted for the delivery of siRNA to the liver^10^.

Some of the novel approaches for delivery of siRNA/miRNA including smarticles for miR-34a delivery *in vivo*^11^, ligand-mediated N-acetyl glucosamine-siRNA (GalNAc) delivery to liver^12^, folate gated miRNA for breast and lung cancer^13^, other lipidic nanoparticles^14^ as a siRNA delivery tactics are currently under preclinical and clinical trials. Recently, the USFDA has approved the lipidic nanoparticle for the delivery of siRNA (Patisiran: Onpattro; by Alnylam Pharmaceuticals) to treat polyneuropathy in patients with hereditary transthyretin-mediated (hATTR) amyloidosis^15,16^. It has been largely advocated that polymeric vectors could offer numerous advantages over the viral vector, for instance, eliminating the off-target effect, prolonged extracellular or serum stability, non-immunogenicity, and easy formulation steps to name the few^17^. Quality-by-design (QbD) driven synthesis of cationic polymeric nano vector (CPNVs), incorporation of the fitting level of crosslinking agents, pH redox-responsive units, osmo-responsive agents, etc. are some of the typical strategies to improve siRNA encapsulation, stabilization and delivery capabilities of polymeric vectors^8^. In an attempt to promote the escape of polymeric vectors from endosomes/lysosomes, moieties that elevate intra-osmotic pressure in endosomes/lysosomes were extensively employed to eventually burst these compartments. However, the premature separation of siRNA from the polymeric vectors in the body fluids, and subsequent degradation by endogenous serum nucleases still stand as the unaddressed challenges^18^.

Several cell-penetrating peptides (CPPs) were developed to increase the extent of cytosolic delivery of siRNAs and other genetic materials^19^. However, despite three decades of research, the fundamental basis for CPP activity remains elusive and non-conclusive. The biomedical application and clinical translation of most of the siRNA delivery devices have been impeded to a great extent due to its poor containment property as well as associated toxicities^20^. To facilitate the intracellular disassembly and facilitate cytosolic release of loaded siRNA, the stimuli-sensitive linkages with the capability to get readily cleaved in response to intracellular acidity or redox conditions were also coupled with PNVs^21^. However, the delivery capability of these hybrid vectors is largely compromised by complex physiological and biological barriers, which restrict their applications in a clinical setting^22^.

Albumin, a natural polymer offers an optimal platform for the development of the drug as well as siRNA delivery vehicles pertain to the countless advantages including abundantly available protein, easy modification, easy purification, non-immunogenic, biodegradable, inertness, encapsulation of hydrophilic payload in the hydrophobic corona and low cost^23^. An albumin-based platform for the delivery of anticancer drug Paclitaxel has already made its way to the market in the form of a USFDA approved product (Abraxane; Celgene Corporation, USA)^24^. Literature suggests that albumin-based nanoparticle selectively binds podocytes of renal bowman’s capsule via FcRn receptor to regulate proteinuria^25^. This makes albumin an advantageous biopolymer for the development of siRNA delivery vector. However, the inherent architectural configuration of albumin is not adequate to guarantee a complete escape of the nanovector from the endo-lysosomal compartment as well as protect the loaded siRNA therapeutics from the harsh endo-lysosomal environment. Further, the anionic carboxylic side chain of albumin imparts limitation towards the encapsulation of anionic siRNA molecules (due to the existence of anionic phosphate side chain in siRNA architect)^26,27^.

**Figure 1:**
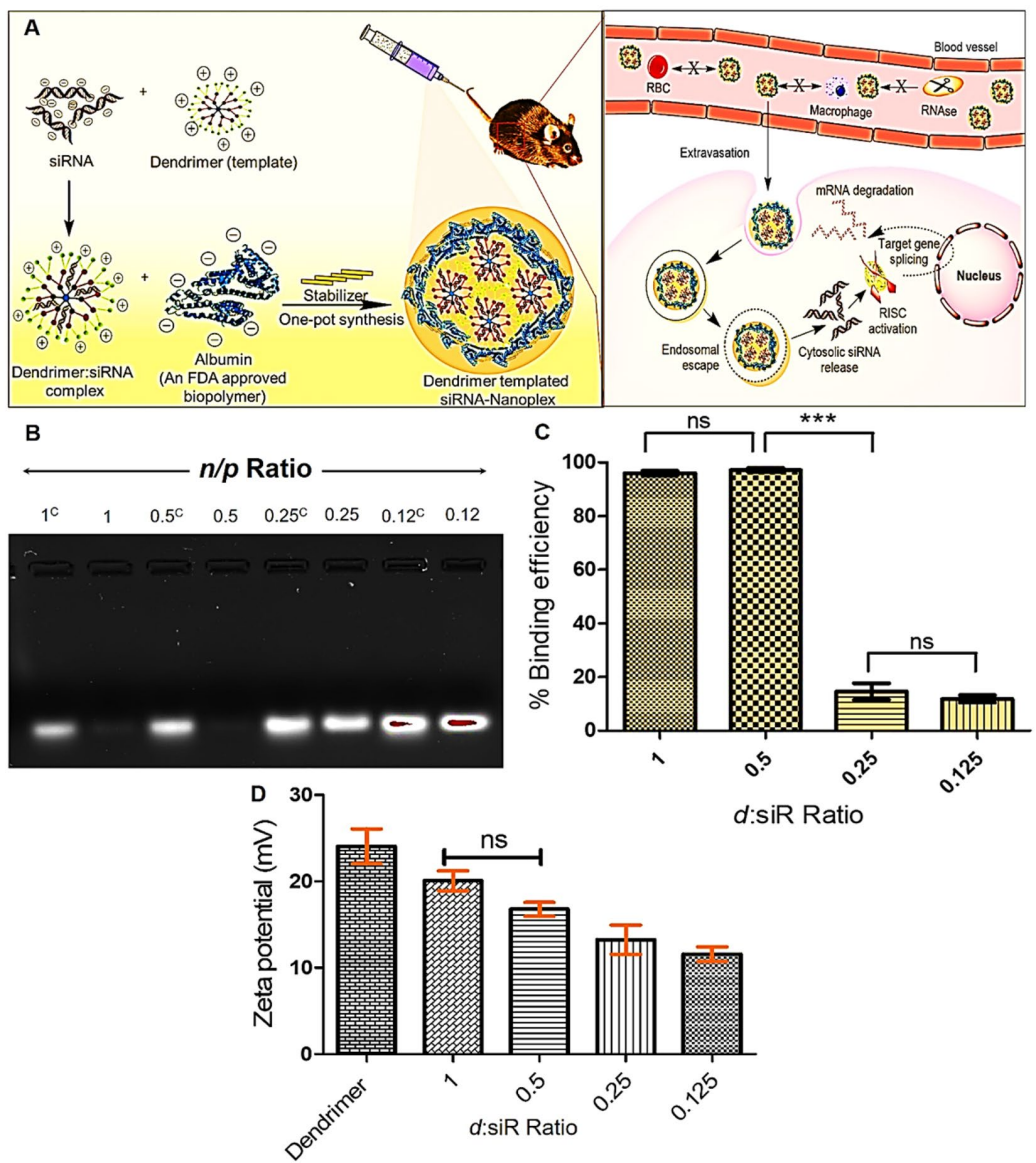
(**A**) Scheme showing encapsulation, stabilization, and delivery of siRNA in Anionic polymeric nanoplex. The developed siRNA-nanoplex imparts serum stability, avoids *in vivo* RNase degradation and mediates its cytosolic delivery following the endosomal escape. The endosomal escape leads to a selective siRNA release of loaded siRNA in the cytosolic region, and this phenomenon was facilitated by strategic incorporation of the dendrimer in the architectural configuration of siRNA-nanoplex. (**B**) Gel electrophoresis for the selection of *d*:siR complexation condition, Here, Lane 1 (1^C^): positive control having siRNA in equivalent amount as in *d*:siR complex prepared at 1 *n/p* ratio; Lane 2: *d*:siR complex prepared at 1 *n/p* ratio; Lane 3 (0.5^C^): positive control having siRNA in equivalent amount as in *d*:siR complex prepared at 0.5 *n/p* ratio; Lane:4: *d*:siR complex prepared at 0.5 *n/p* ratio; Lane 5 (0.25^C^): positive control having siRNA in equivalent amount as in *d*:siR complex prepared at 0.25 *n/p* ratio; Lane 6: *d*:siR complex prepared at 0.25 *n/p* ratio; Lane 7 (0.12^C^): positive control having siRNA in equivalent amount as in *d*:siR complex prepared at 0.12 *n/p* ratio; Lane 8: *d*:siR complex prepared at 0.12 *n/p* ratio (**C**) Binding efficiency of *d*:siR complex, (**D**) surface zeta potential of *d*:siR formed using *n/p* ratio of 1, 0.5, 0.25, and 0.125. Results are represented as mean ± S.D. *(n* = 3*)*.

Histone deacetylases (HDACs) have been implicated in podocyte dysfunction to mediate the onset and development of diabetic nephropathy (DN)^28,29^. The development of HDAC4-specific gene inhibitors may provide an efficacious therapeutic tool for DN. Hence, in this study, we aim to deliver HDAC4 siRNA using the innovative siRNA delivery tool developed by us as a proof of concept. We evaluate and report it’s *in vitro* and *in vivo* HDAC4 gene silencing capability using podocytes as well as in DN mouse model. The core goal of this study was to develop and test the siRNA delivery vehicle that can offer *in vivo* stabilization, overcome endosomal degradation, and can mediate intracellular siRNA delivery (Fig. 1A). For proof of concept; we employed HDAC4 siRNA in the treatment of DN, however, the reported technology can be extended to other gene therapeutics viz miRNA, plasmid, etc. to name the few.

## Results

### Screening of Dendrimer/siRNA (*d*:siR) complex

The binding efficiency of siRNA with the polycationic dendrimer template was investigated by determining the band binding efficiency and band intensity of the siRNA (Fig. 1B,C). The results infer that at a *surface to charge ratio (n/p)* of 1 and 0.5, the binding of siRNA with dendrimer template was 94.78 ± 1.07% and 90.50 ± 0.93%, respectively. The binding of siRNA with dendrimer template was well complemented by the absence of migration of free siRNA in the gel electrode compared to other *n/p* ratios. On the other hand, upon reducing the *n/p* ratio to 0.25 or 0.125, the binding efficiency significantly reduced to 30.25 ± 2.85% (*p* < 0.001) and 18.33 ± 19.26% (*p* < 0.001), respectively. The ineffective siRNA binding at *n/p* ratio below 0.5 can be seen by the emergence of band intensities corresponding to free/uncomplexed siRNA. This suggests that a specific *d*:siR composition and *n/p* ratio (*d*:siR: *n/p* ratio 0.5) is required to form an effective *d*:siR complex Fig. 1B,C.

### Net Surface zeta potential of *d*:siR complex

The surface zeta potential of *d*:siR complex formed at various *n/p* ratio was determined (Fig. 1D). The free dendrimer bears a net positive surface zeta potential of +24.04 ± 3.52 mV, which upon complexation with siRNA reduced to +20.06 ± 2.00 mV, +16.76 ± 1.37 mV, +13.24 ± 2.91 mV, and +11.57 ± 1.45 mV, respectively of *d*:siR formed using *n/p* ratio of 1, 0.5, 0.25, and 0.125, respectively.

### Quality-by-Design (QbD) driven synthesis and characterization of siRNA Nanoplex

**Figure 2:**
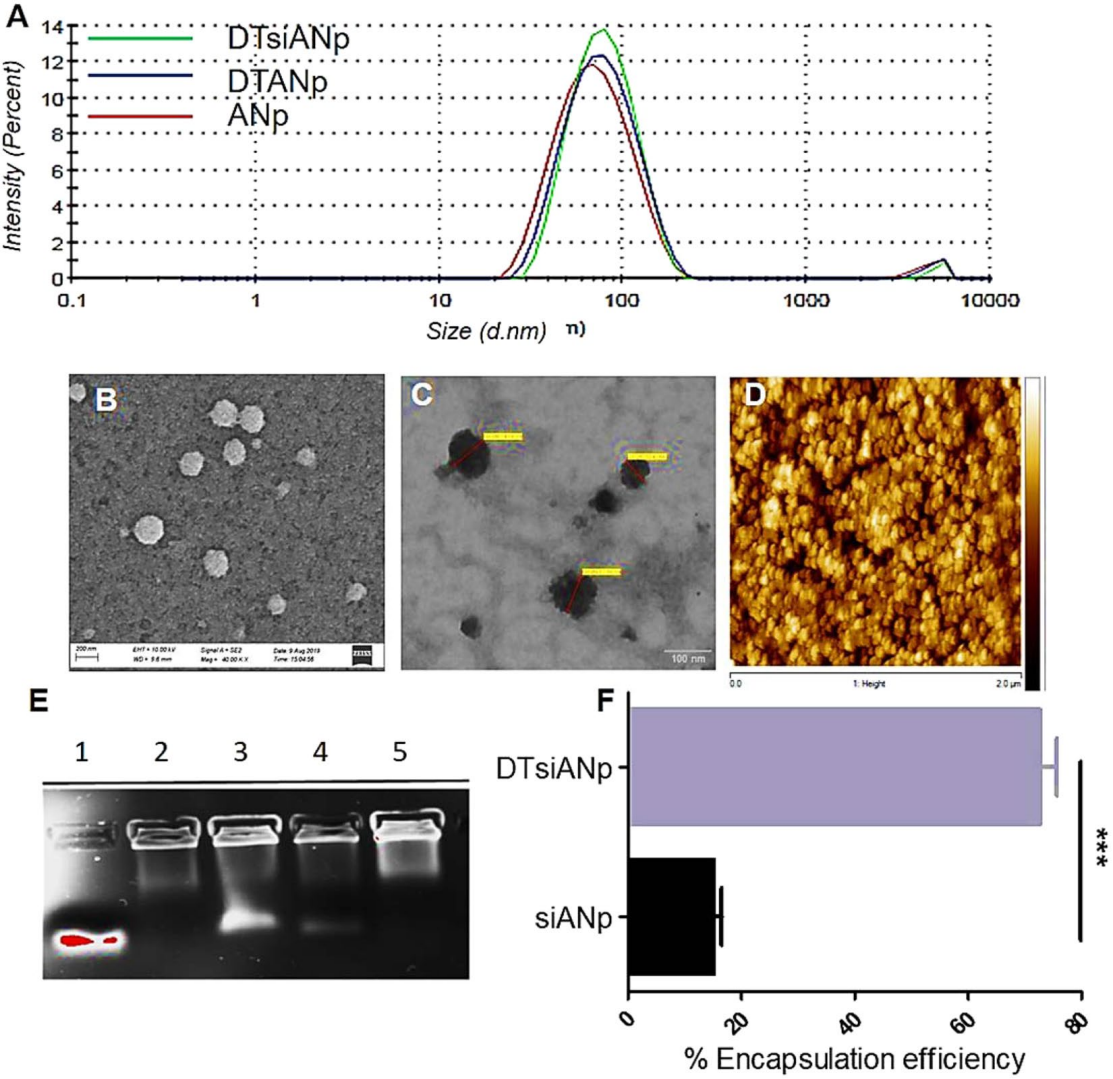
(**A**) Hydrodynamic particle size distribution of ANp, DTANp, and DTsiANp using DLS, (**B**) SEM image of DTsiANp (**C**) TEM of DTsiANp, (**D**) AFM of DTsiANp (**E**) Gel electrophoresis, Lane 1: naked siRNA, Lane 2: siANp after centrifugation, Lane 3: siANP after centrifugation (supernatant), Lane 4: DTsiANp after centrifugation (supernatant), Lane: 5 DTsiANp after centrifugation, (**F**) encapsulation efficiency of siRNA in siANp and DTsiANp determined using Ribogreen assay (***p < 0.0001). Results are represented as mean ± SD *(n* = *3)*.

The purity of albumin was assessed via SDS-PAGE (Fig. S9), BCA assay (≥96%; Fig. S10), and MALDI-TOF/MS (Fig. S11) for the evaluation of any other component from the fraction V. The synthesis process design and process parameters were optimized using the Box-Banken QbD approach to produce siRNA loaded Nanoplex (siANp) and dendrimer templated siRNA nanoplex (DTsiANp) (Target particle size: ≤70 nm). The blank nanoplex counterparts including ANp and DTANp were also produced following the same protocol for comparison (*refer supporting information*). The dynamic light scattering (DLS), scanning electron microscopy (SEM), transmission electron microscopy (TEM), and atomic force microscopy (AFM) was performed to characterize the properties of siRNA nanoplex for hydrodynamic particle size, polydispersity index (PDI), surface zeta potential (ζ, mv), and surface topography morphology. DLS suggested siANp and DTsiANp be of nanometric size with a hydrodynamic particle size of 66.93 ± 2.90 nm (ζ, −26.8 ± 0.89 mV; PDI: 0.210 ± 0.01I) and 64.51 ± 0.83 nm (ζ, −16.1 ± 1.06 mV; PDI: 0.187 ± 0.06), respectively (Fig. 2A). The representative TEM, SEM and AFM images of DTsiANp showed the nanoplexes to be nanometric, rounded-oval with smooth surface topography (Figs 2B–D; S16). The AFM analysis suggested nanoplex to be aggregates of spherical particles of ~30 nm in diameter. It may be noted that the nanoplex size observed by AFM for ANp, DTANp, siANp, and DTsiANp found to be smaller than the ones recorded via DLS technique. This is probably due to the shrinkage of the nanoparticles during the drying process employed during the sample preparation for AFM imaging analysis (Fig. S17). These outcomes are in agreement with reported literature on the size of albumin nanoparticles^30^.

After characterization of nanoplex, the assays were also done to confirm the presence of albumin and dendrimer inside the nanoplex. It was found that albumin remained intact after nanoplex preparation and forms the major component of the nanoplex system (~95%; Fig S10 and S12). The presence of dendrimeric template in the nanoplex was confirmed via TNBSA assay. It observed that number of the primary amino group got significantly enhanced in DTsiANp (22.54 ± 1.67%; *p* < 0.05) and DTANp (23.11 ± 1.08%; *p* < 0.05) after the incorporation of the dendrimeric template as compared to the siANp and ANp (Fig. S13). The incorporation of dendrimeric template in nanoplex was further confirmed by zeta potential analysis (Fig. S14).

### The siRNA encapsulation efficiency of Nanoplex

The encapsulation efficiency of siRNA nanoplex was determined using gel retardation assay and re-verified by Ribogreen assay. The quantitation of gel showed the presence of approximately 18.51 ± 4.07% naked siRNA following gel electrophoresis of DTsiANp, as against to almost 89.21 ± 7.05% naked siRNA as observed (Fig. 2E) in case of siANp (*p* < 0.05). This encapsulation of siRNA in nanoplex was also assessed by Ribogreen assay, and the outcomes were analogous to the results obtained by gel retardation assay. The siRNA encapsulation efficiency in DTsiANp and siANp was found to be 72.62 ± 3.01% and 14.90 ± 1.53%, respectively (Fig. 2F). The result infers that the siRNA encapsulation efficiency in albumin nanoplex gets significantly enhanced while adopting the dendrimer templated approach. Herein, the siRNA encapsulation efficiency enhanced by 3.87 ± 1.04 fold (*p* < 0.0001) as compared siANp. Further, the actual encapsulation of siRNA in nanoplex was confirmed using RNase protection assay. This assay was also performed to verify that the siRNA exists inside the nanoplex and is not the mere precipitate of siRNA and the biopolymer. The outcome of this investigation confirmed that the siRNA exists inside the nanoplex as encapsulated form rather than simple precipitation of siRNA (Fig. S15).

### Serum stability of siRNA Nanoplex

**Figure 3:**
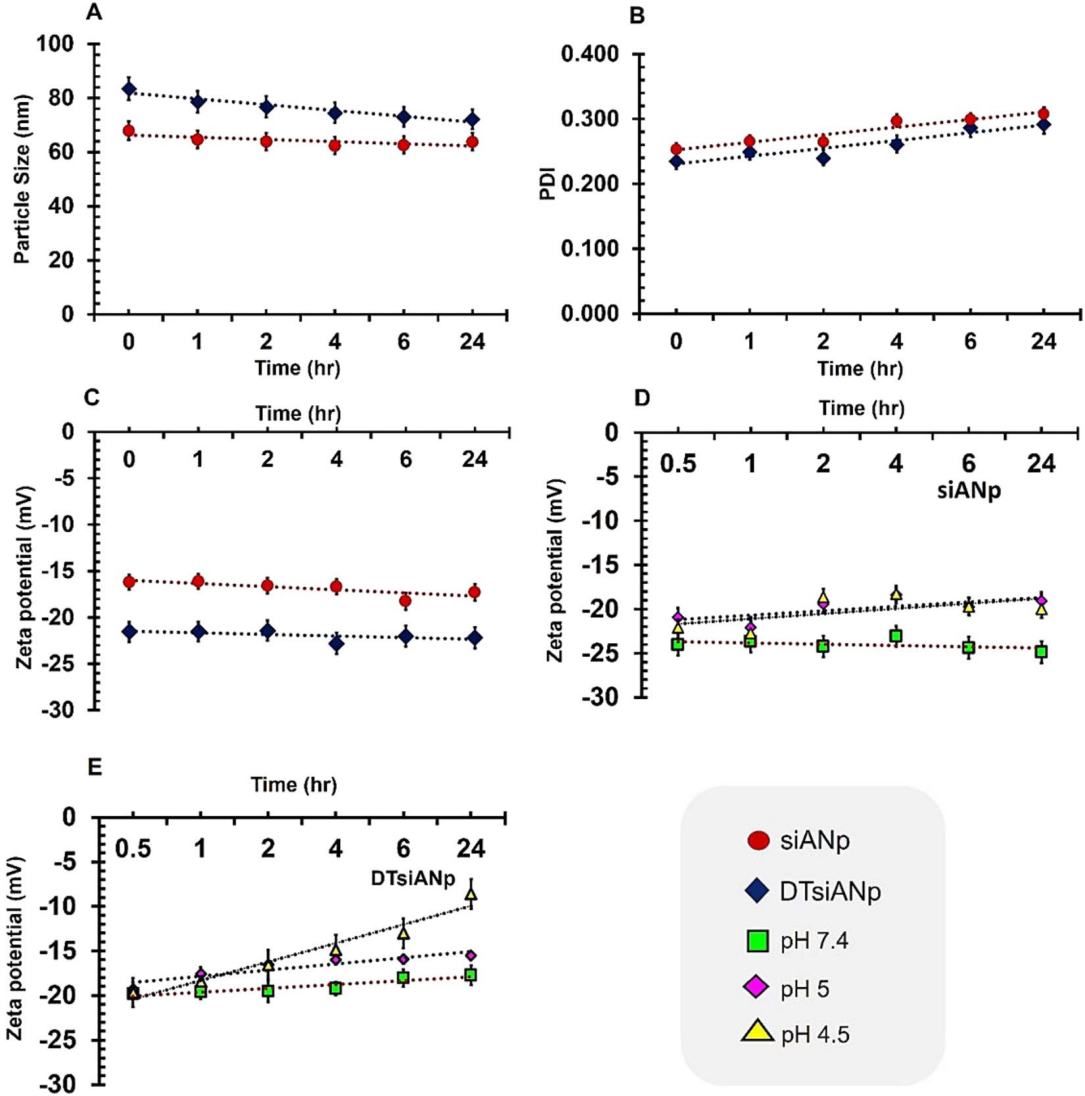
Illustration of serum stability of siANp and DTsiANp in terms of (**A**) hydrodynamic particle size (**B**) PDI (**C**) surface zeta potential. pH responsiveness of nanoplexs in terms of surface zeta potential (**D**) siANp, (b) DTsiANp at pH 7.4, 5.5 and 4.5. Results are represented as mean ± S.D. *(n* = *3)*.

**Figure 4:**
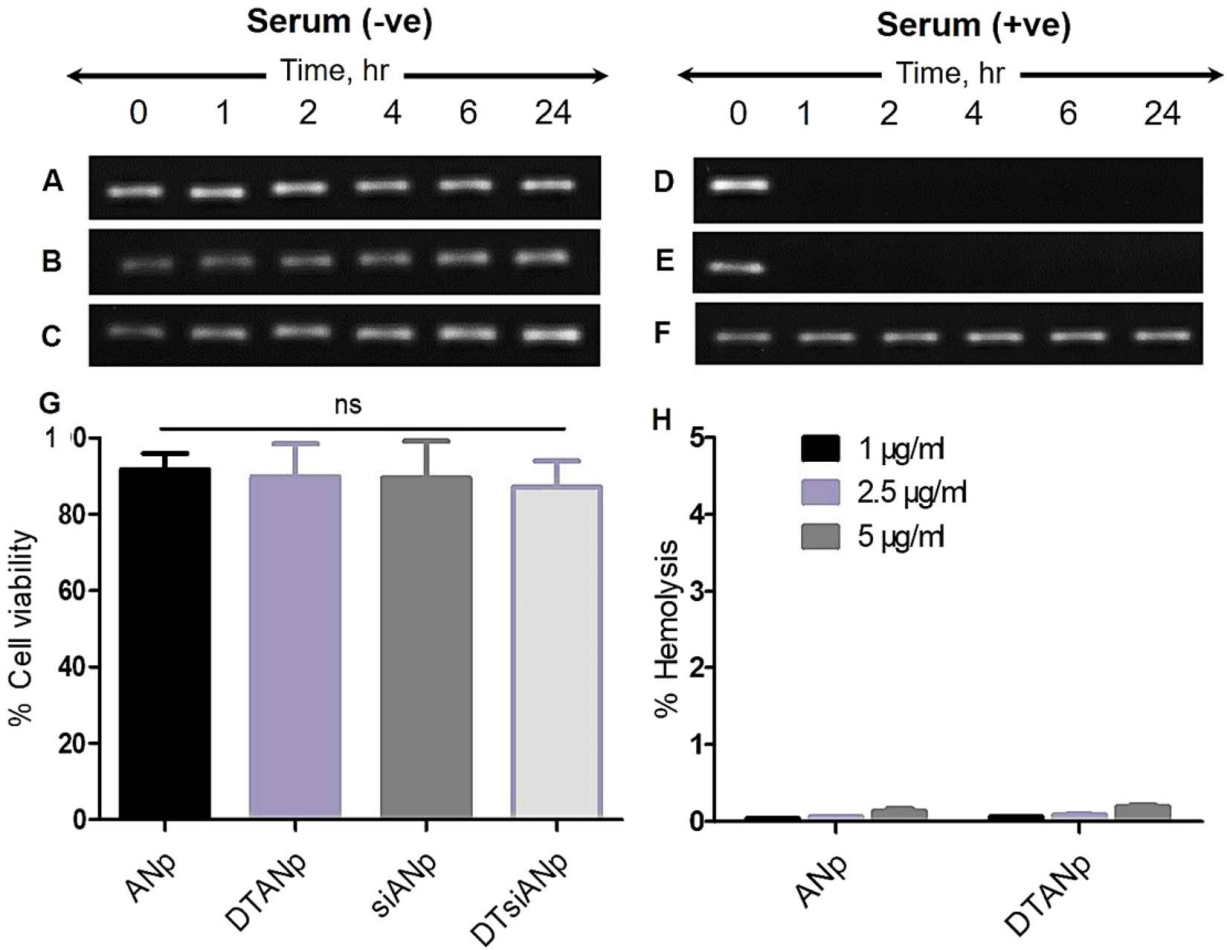
Stability profile of siRNA in absence of serum as evaluated by gel electrophoresis (**A**) naked siRNA, (**B**) siANp, (**C**) DTsiANp. Stability profile of siRNA in presence of serum as evaluated by gel electrophoresis (**D**) naked siRNA, (**E**) siANp, (**F**) DTsiANp. (**G**) MTT assay for percentage cell viability of ANp, DSAN, siANp, and DTsiANp in HG (30 mM) exposed podocytes cells (**H**) Hemo-compatibility assay for ANp and DTANp. Results are represented as mean ± S.D. (*n* = *3*).

The outcome of this investigation suggested that in presence of serum, there was an insignificant change in the particle size, PDI and zeta potential of siANp and DTsiANp even after 24 hr (Fig. 3A–C; *p* > 0.05). It may be noted that naked siRNA exposed to the serum is highly prone to undergo degradation by serum RNase enzyme (Fig. 4A–F). Hence, the capability of siANp and DTsiANp to protect siRNA against serum RNase enzyme was investigated by gel electrophoresis assay. Here, at zero time point (serum untreated), the siRNA bands were remained intact and considered as starting control for succeeding time points. It was found that in the presence of serum, naked siRNA (Fig. 4D) degrades completely in less than 1 hr incubation time. While in the case of siANp, the siRNA was degraded and was not able to remain stable in the presence of serum due to surface-bound siRNA on siANp (Fig. 4E). On the other hand, DTsiANp (Fig. 4F), showed retention of siRNA band in the presence of serum till 24 hr. The percentage siRNA stability in the presence of serum was further confirmed through percentage siRNA band intensity with reference to siRNA band intensity of serum untreated group (Fig. S8). It was found that the siRNA remains stable in DTsiANp, which can be ascribed to the presence of dendrimeric template in this formulation. Because of the dendrimeric template, siRNA does not easily come in contact with the serum and hence remain stable until a prolonged period of time (>24 hr).

### Endo/Lysosomal Escape tendency of developed siRNA Nanoplex

The pH sensitivity of nanoplexes and their ability to undergo physicochemical and morphological amendments inside the endo-lysosomal compartment was assessed under pH 5.5, pH 4.5 and pH 7.4. Here, pH-dependent protonation behavior of siANp (conventional plain albumin-based nanoplex) compared to DTsiANp (nanoplex that contain dendrimer) was evaluated. A significant change in the hydrodynamic particle size, PDI and sur\face charge was noted (*p* < 0.05) when the pH of the incubation milieu was changed from physiological pH to endosomal (acidic) environment. An insignificant change in the particle size and zeta potential of siANp (particle size: 0.084-fold change; ζ: 2.65 ± 1.98% change) and DTsiANp (particle size: 0.062-fold change; ζ: 1.2 ± 1.32% change (*p* > 0.05)) was observed following their incubation in physiological pH 7.4 inferring their stability under physiological condition (Figs S4; 3D,E).

The incubation of siANp under pH 7.4, 5.5 and 4.5 did not elicit any change in its effective particle size. However, after incubation of DTsiANp under acidic environment (pH 5.5; early endosomal pH) and under pH 4.5 (late endosomal pH), a significant enhancement in their particle size was observed. Here, the particle size of DTsiANp increased by 41.98 ± 2.47% (under pH 5.5; *p* < 0.001) and 74.14 ± 0.41% (under pH 4.5; *p* < 0.001) (Fig. S4).

In addition, surface zeta potential also affected significantly upon a change in pH (Fig. 3D,E; *p* < 0.05). In case of, plain albumin, the surface zeta potential was not significantly increased in endosomal pH 5.5 (17.92 ± 2.06%; *p* > 0.05) and in pH 4.5 (15.55 ± 1.92%; *p* > 0.05) over pH 7.4 (15.11 ± 0.93%; *p* > 0.05). Whereas, in case of the plain dendrimer, the zeta potential was enhanced significantly by 24.05 ± 1.01% (pH 5.5; *p* < 0.05) and 41.41 ± 1.67% (pH 4.5; *p* < 0.05) as compared to pH 7.4 (11.77 ± 1.72%) (Fig. S18). DTsiANp surface charge was enhanced to 19.36 ± 1.15% (pH 5.5; *p* < 0.05) and 56.31 ± 1.84% (pH 4.5; *p* < 0.01) compared to pH 7.4 (−14.9 ± 1.26 mV). Suggestively, the results infer the pH-responsive morphological changes in the architect of DTsiANp owing to the protonation of free primary amines of dendrimeric template in acidic pH environment.

The Endo/lysosomal escape effect of nanoplex was also evaluated by means of lyso-tracker red dye. Results (Fig. S19) suggested that DTsiANp treated cells were showed significant yellow fluorescence due to co-localization of green (FAM-siRNA) and red fluorescence (endosome selective lyso-tracker red) after 8 hr. Initially, at 6 hr there was very less red fluorescence was observed in comparison to 8 hr and suggesting endosomal uptake of DTsiANp. At 12 hr, reduction in the red fluorescence and parallel enhanced green fluorescence was appeared in the cytosolic region, which infers the endosomal escape ability of DTsiANp. Whereas, siANp was not exhibiting any fluorescence even at 12 hr.

### Biocompatibility of Nanoplex

MTT assay was employed to evaluate the biocompatibility of developed nanoplexes viz: ANp, DTANp, siANp, and DTsiANp in normal as well as in high glucose (30 mM) HG-treated podocytes DN model. The results inferred that all prepared nanoplexes were biocompatible with approximately 100% cellular viability of podocytes (Fig. 4G).

### Hemocompatibility assay

As illustrated in Fig. 4H, all the prepared nanoplexes were found to be highly hemocompatible. The ANp and DTANp showed less than 0.2% hemolysis (*p* > 0.05) when tested under all possible clinically applicable concentrations (1 μg/ml, 2.5 μg/ml and 5 μg/ml) inferring endogenous albumin-based ANp and DTANp to be safe and biocompatible with the blood compartment.

### Cellular uptake of siRNA nanoplex in HG-treated podocytes DN model

**Figure 5:**
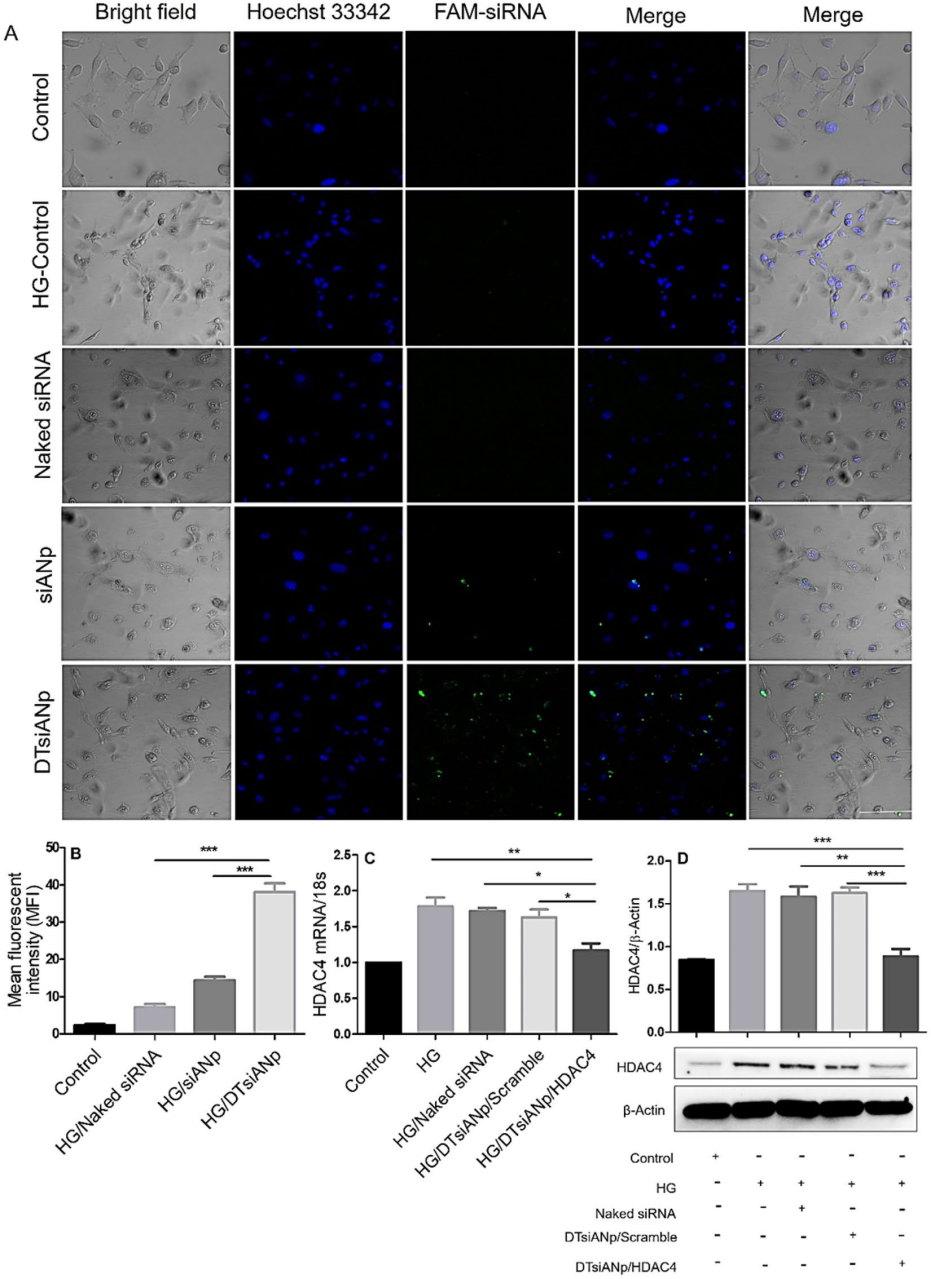
(**A**) Confocal microscopic image of FAM-siRNA cellular uptake in HG (30 mM) treated podocyte DN model following treatment of naked siRNA, siANp and DTsiANp. (**B**) Mean fluorescence intensity of FAM-siRNA (**C**) HDAC4 silencing efficiency by qRT-PCR in HG-treated podocytes, *p < 0.05 vs. DTsiANp/Scramble, *p < 0.05 vs. Naked siRNA, **p < 0.01 vs. HG treated *p*odocytes (**D**) western blot analysis for HDAC4 protein expression in HG-treated podocytes. HG-treated cells were taken as a positive control. Western blot analysis was done using ImageJ (NIH, Bethesda, MD). Results are represented as mean ± SD, *(n* = *3)*. *indicates *p* < 0.05. Scale bar: 50 µm; Blue fluorescence: Hoechst 33342 (Bisbenzimide), green fluorescence: FAM-siRNA (6-carboxyfluorescein).

The cellular uptake of naked siRNA, siANp, and DTsiANp was performed in HG-treated podocytes DN model^31^ and investigated by confocal laser scanning microscopy (CLSM). Here, HG treated podocytes cells showing morphological chages such as cellular membrane blebbing, reduction in cellular volume and rounding of the cell^32^. The results (Fig. 5A) suggested that DTsiANp gets internalized inside the cell in 12 hr as observed by the higher FAM-siRNA associated green fluorescence inside the cell as compared to naked siRNA and siANp. The cellular uptake of DTsiANp was found to be 4.45-fold and 1.63-fold higher mean fluorescence in podocytes cells as compared to naked siRNA and siANp (Fig. 5B).

### *In vitro* gene silencing efficiency of siRNA nanoplex in HG-treated podocytes DN model

In HG condition, HDAC4 gene primarily contributes to podocytes injury in DN^33^ and hence, HDAC4 gene was selected as a target for testing the siRNA delivery system as proposed in this investigation. qRT-PCR was performed to evaluate the expression level of HDAC4 gene at a transcriptional to recognize the molecular mechanism of its therapeutic effect. The HDAC4 gene was found to be significantly overexpressed in HG treated podocytes, which is in agreement with existing reports^33^. The HG treated podocytes were then treated with naked siRNA, DTsiANp/HDAC4, and DTsiANp/scramble for 24 hr. Naked siRNA treated podocytes showed an insignificant suppression of HDAC4 expression (3.5 ± 0.93%, *p* > 0.05). Whereas, DTsiANp/HDAC4 treated cells showed 28.39 ± 1.37% (*p* < 0.05) repression of HDAC4 mRNA expression compared to DTsiANp/Scramble. A 32.11 ± 1.87% (*p* < 0.05) and 34.51 ± 1.87% (*p* < 0.01), respective downregulation of HDAC4 mRNA was noted following the treatment of DTsiANp/HDAC4 as compared to naked siRNA and HG treated podocytes (Fig. 5C). This suggests efficient silencing of dysregulated HDAC4 in HG treated podocytes cells by DTsiANp. Further, the effect of gene silencing on HDAC4 protein was also evaluated by western blot analysis.

### Evaluation of HDAC4 protein expression in siRNA nanoplex treated HG-podocytes DN model

HDAC4 is one of the centrally acting biomolecules involved in the functioning of podocytes. In HG condition, overexpression of HDAC4 induces the inflammation, apoptosis, and autophagy in podocytes^33^. In line with the reported literature, we also observed an enhanced HDAC4 protein expression in HG treated podocytes^33^. The treatment of naked siRNA to HG treated podocytes cells did not exhibit significant downregulation (4.02 ± 0.90%; *p* > 0.05) in the relative HDAC4 protein level. In agreement with the qRT-PCR outcomes, relative HDAC4 protein expression was found to be significantly suppressed following the treatment of DTsiANp/HDAC4 by 44.24 ± 2.10% (*p* < 0.01) and 45.56 ± 3.61% (*p* < 0.001) as compared to the naked siRNA and DTsiANp/scramble, respectively (Fig. 5D). Further, DTsiANp/HDAC4 successfully repressed the relative HDAC4 protein level (46.27 ± 3.96%; *p* < 0.001) in HG treated podocytes model.

### Effect of siRNA nanoplex treatment on metabolic parameters in C57BL/6 DN mice model

**Figure 6:**
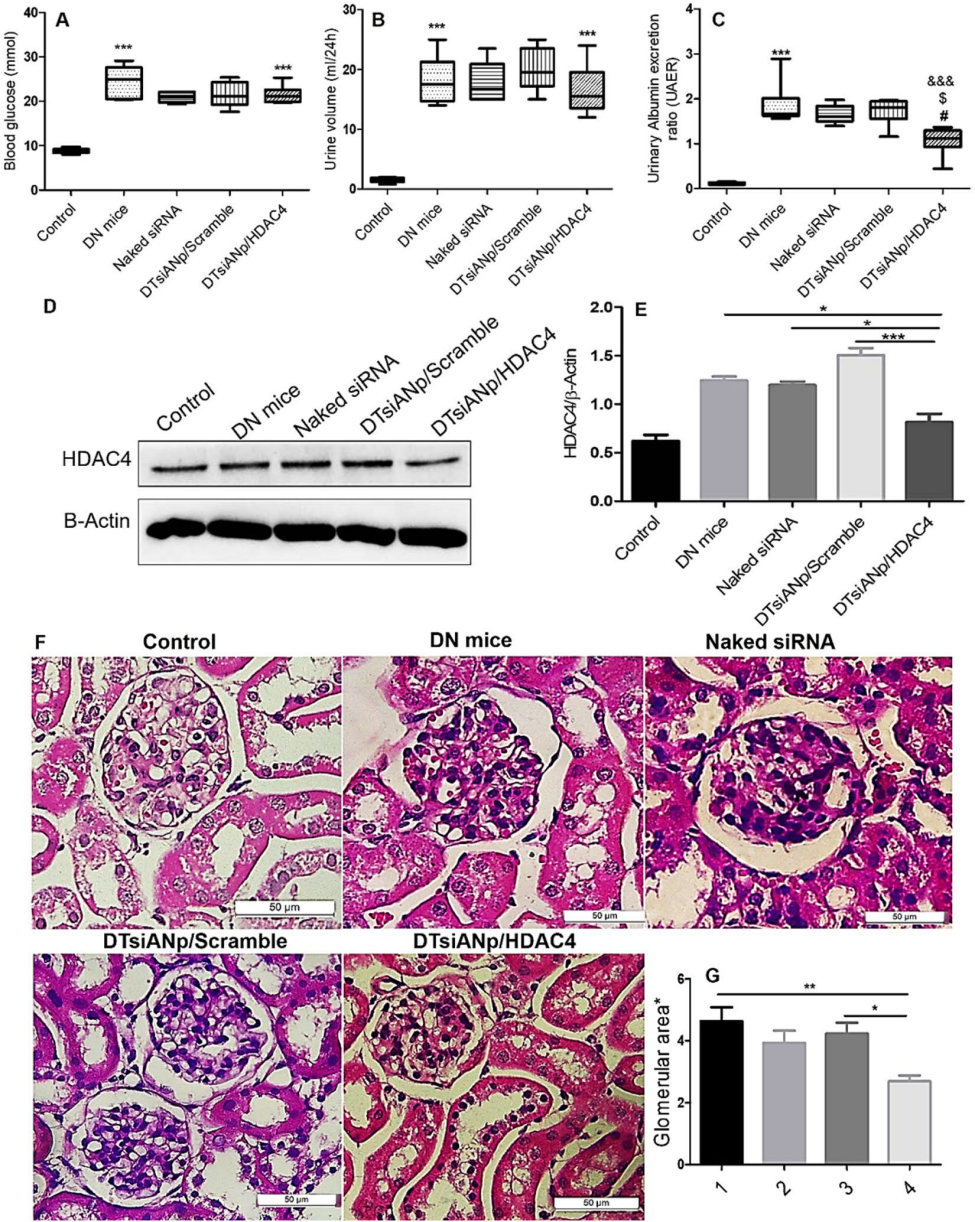
(**A**) Mice glucose level, (**B**) urine volume (**C**) Urinary albumin excretion ratio (UAER) in control, DN mice, and treated groups (naked siRNA, DTsiANp/Scramble and DTsiANp/HDAC4), ^#^*p* < 0.05 vs. naked siRNA, ^$^*p* < 0.05 vs. DTsiANp/Scramble, ^&&&^*p* < 0.001 vs. DN mice, ***p < 0.0001; control vs. DN mice (**D**) HDAC4 protein expression in healthy control and DN diseased kidneys as detected by western blot analysis, (**E**) protein levels in kidneys of mice post-treatment. Relative quantification was normalized by β-actin expression *p < 0.05 versus DN mice, ***p < 0.001 vs. DN mice treated with DTsiANp/scramble, *p < 0.05 versus DN treated with naked siRNA (**G**) Histological studies of the kidney after 4-week treatment (**F**) glomerular area (*ratio healthy control group) 1; DN mice, 2; Naked siRNA, 3; DTsiANp/Scramble, 4; DTsiANp/HDAC4. **p < 0.01 vs. DN mice. Results are represented as mean ± SEM of 5–8 mice/group.

As shown in Fig. 6A–C, the glucose level, urine volume, and urinary albumin excretion ratio (UAER; representative of severity of proteinuria) was found to be markedly increased in DN induced mice group (*p* < 0.05) as compared to control (healthy mice) group. The four-week treatment of DTsiANp/HDAC4 in DN mice group did not reduced the urine volume and glucose concentration significantly as compared to the DN mice group treated with the naked siRNA and DTsiANp/Scramble (Fig. 6B). Further, it was observed that the naked siRNA was not significant in urine volume and glucose concentration as compared to vs. DTsiANp/Scramble (*p* > 0.05). The UAER was found to be extremely low in the healthy control group (0.113 ± 0.02), which severely got amplified in DN diseased mice (1.80 ± 0.56; in 4 weeks; *p* < 0.001). In case of DTsiANp/scramble and naked siRNA treated group, UAER was found to be 1.72 ± 0.29 (*p* > 0.05) and 1.65 ± 0.20 (*p* > 0.05), respectively which was found similar as shown in DN mice group. However, upon treating the DN induced mice with DTsiANp/HDAC4, significant repression was observed in the UAER at 4 weeks as compared to DN disease group (1.07 ± 0.30; *p* < 0.01 vs DN mice, *p* < 005 vs. DTsiANp/Scramble, *p* < 0.05 vs. naked siRNA; Fig. 6C).

### *In vivo* Evaluation of HDAC4 protein expression in siRNA nanoplex treated C57BL/6 DN mice model

The HDAC4 protein expression was evaluated by western blot analysis following isolation of protein from the cortical region of kidneys from healthy control, streptozotocin-induced DN mice group and nanoplex treated groups (DTsiANp/HDAC4, DTsiANp/scramble, naked siRNA (Fig. 6D,E). A significantly enhanced HDAC4 protein (1.0 ± 0.089-fold) was observed in DN mice control group as compared to the healthy control group. The treatment of DN induced mice with naked HDAC4 siRNA leads to a slight downregulation of HDAC4 protein expression by 3.90 ± 0.95% (*p* > 0.05). The treatment of DTsiANp/Scramble to DN induced mice elicited no protective effect and showed a significant rise in relative HDAC4 protein level by 1.5 ± 0.12-fold as compared to healthy control. The treatment of DTsiANp/HDAC4 in the DN mice led to a significant reduction in the relative expression of HDAC4 protein by 31.87 ± 1.13% (*p* < 0.05) compared to naked siRNA and 45.87 ± 2.01% (*p* < 0.001) compared to DTsiANp/scramble (Fig. 6E).

### Renal histopathological analysis in siRNA nanoplex treated C57BL/6 DN mice model

The renal histological changes in the kidney tissue sections of C57BL/6 DN mice stained with hematoxylin-eosin is shown in Fig. 6F. The figure revealed the presence of damaged glomerulus of kidneys of DN mice. Further, the kidney sections also suggested the existence of significant masses of accumulated mesangial matrix or hyper proliferated mesangial cell and glomerular sclerosis as compared to the control healthy mice group (Fig. 6F–G). It was confirmed through an increased glomerular area ratio by about 4.63 ± 1.35 (*p* < 0.001) of DN over healthy control. Naked siRNA (3.93 ± 1.24 ratio; *p* > 0.05) and DTsiANp/scramble (4.23 ± 1.10; *p* > 0.05) treated mice showed an insignificant change in the glomerular area ratio compared to DN mice group. After 4-week treatment with DTsiANp/HDAC4 glomerular ratio was found 2.69 ± 0.61 (*p* < 0.001 vs. DN mice and *p* < 0.05 vs. DTsiANp/scramble). These morphological changes in terms of the mean ratio of glomerular area were reduced significantly after treatment of DTsiANp/HDAC4 by 36.49 ± 2.18% (*p* < 0.05) (Fig. 6G) over DTsiANp/scramble treated DN mice.

## Discussion

With years of appreciable research data support, therapeutic potency, as well as a winning Noble Prize of Medicine (Award year 2006; Sir Craig Mello @ Sir Andrew Fire), has greatly enhanced the expectation with RNAi therapeutics^1^. However, due to the lack of an apt siRNA delivery system, the science has greatly stagnated or progressing sluggishly in context to their movement from lab-to-clinic translation^5^. Numerous efforts have been made to come up with an apt siRNA delivery vector with superior delivery attributes, commercial viability, safety as well as high clinical translation capability^17^. There was some extent of success with viral vectors and lipidic transfecting reagents, however, either the safety concerns or the lack of *in vivo* fittingness discourages their clinical utilization. In this meadow, cationic polymeric systems were extensively utilized, however, the toxicity issues associated with them substantially impedes the further development^34,35^.

Herein, we report a novel, simple and clinically translatable approach for the encapsulation, stabilization, as well as safe delivery of siRNA using a biocompatible and FDA-approved albumin carrier. It may be noted that the conventional albumin-based siRNA delivery vector was found insufficient, incapable of encapsulating as well as effective homing of siRNA payload in the cytosolic compartment. This can be ascribed to the mismatch in their physiochemical attributes, wherein both being negatively charged^27^. At the same time, conventional albumin siRNA vector lacks the capability to avoid premature siRNA release in blood as well as mediate endo/lysosomal escape in the cellular compartment leading to its substantially reduced efficacy^22^. This scientific report presents a simple protocol based on the unpretentious and scientifically acclaimed phenomenon that can transform albumin to a resourceful biopolymer for siRNA delivery.

The siRNA effectively forms the complex with dendrimer template owing to the existence of opposite electrostatic charges on them^8^. Depending upon the associated *n/p* value, the *d*:siR complex of varying net charge was formed ranging from +24.04 ± 3.52 to +11.57 ± 1.45 mV. Our overall goal was thus to develop a *d*:siR complex of net positive charge and then develop a simple process to load siRNA inside an anionic albumin biopolymer. Hence, at first instance, our target was to develop *d*:siR complex that stably complex siRNA and avoid its degradation. For this, we strategically prepared *d*:siR at varying *d*:siR *n/p* ration and found that at *d*:siR *n/p* value of >0.5 complexation between siRNA and dendrimer was most firm and complete. During gel electrophoresis, the positively charged dye, ethidium bromide can bind with negatively charged siRNA, if available freely and accessible to give proportionate band in Gel Doc instrument (Bio-Rad, USA).

It was found that the *d*:siR forms a stable and viable complex at *n/p* value of 1 and 0.5 as evinced by the inability of ethidium bromide to approach as well as bind siRNA (Fig. 1B,C). From this experiment, we selected *d*:siR *n/p* ration of 0.5 considering the insignificant difference in the property and quality of resulted *d*:siR complex.

The resultant *d*:siR complex was encapsulated inside albumin biopolymer to form siRNA nanoplex (DTsiANp; 64.51 ± 0.83 nm; ζ, −16.1 ± 1.06 mV; PDI: 0.187 ± 0.06). A QbD approach was adapted to attain DTsiANp of average hydrodynamic particle size ≤70 nm. It may be noted that the glomerular fenestrations bear the effective pore size of 70 to 100 nm. A wide array of available literature suggests nanoplex of target size ≤70 nm does not get filtered from the renal pathway but gets passively localized inside the kidney glomerular fenestrations^36–38^. The formed DTsiANp showed an appreciable siRNA encapsulation efficiency of ~75% as confirmed through gel retardation and Ribogreen assay as compared to the nanoplex formed without dendrimer template approach (~15%; classical approach using plain albumin; Fig. 2F).

A prime challenge in *vivo* administration of siRNA lies in their degradation by circulatory RNase enzymes^39^. Hence, the prime requirement of an ideal nanoplex is to ameliorate the encapsulated siRNA as well as prevent its *in vivo* RNase degradation. To deduce this ability, a serum stability study profile of developing nanoplex was studied. It may be noted that the serum interaction of nanoplex comes with an adjusted alternation in the morphology, particle size as well as a surface charge of the nanocomplex. After incubating the developed nanoplexes with the serum, an insignificant change in the morphology, effective particle size, PDI as well as surface zeta potential was noticed (Fig. 3A–C). This infers the inertness of the nanoplex towards the serum, which can be ascribed to the omnipresence of albumin skeleton in the nanoplex. Albumin is a natural biopolymer with its wide availability in the body as well as is known for its inertness^38^.

The naked siRNA exposed to the serum was highly prone to undergo degradation by serum RNase enzyme due to the direct accessibility of siRNA with serum (Fig. 4D). Hence, the capability of developed nanoplexes to protect the encapsulated siRNA against serum RNase enzyme was investigated following its gel electrophoresis. Notably, it was found that the siANp lacks the ability to protect siRNA from serum RNase enzyme due to availability of siRNA in loosely bound form. This can be ascribed to the loose and open architect of siANp that makes it directly accessible to the serum RNase enzyme and siRNA was easily degraded within 1 hr under serum condition (Fig. 4E). This also supports our original notion regarding the insufficiency of conventional albumin nanoplex in siRNA delivery, and our concept to bring innovation to albumin architect to make them siRNA delivery capable. The dendrimer templated encapsulation of siRNA in DTsiANp protects the encapsulated siRNA from serum RNase enzyme degradation. Due to the dendrimeric template, the formed DTsiANp gains an electrostatically stable architect as evinced by the stability study. The tight electrostatically stable architect of DTsiANp protects the encapsulated siRNA from coming in direct contact with the serum RNase enzyme and hence protect it from serum degradation (Fig. 4F). Therefore, it can be stated that DTsiANp is capable enough to hold and protect the encapsulated siRNA from serum endonucleases enzyme for more than 24 hr.

Another foremost challenge with siRNA lies in their degradation in the endo-lysosomal compartment. Hence, one of the overarching goals of this research to develop a nanoplex with the capability to escape from the acidic endo-lysosomal compartment. The pH-dependent stability of developed nanoplex was performed at pH 7.4 (to assess physiological stability), pH 5.5 (effect of early endosomal pH), and pH 4.5 (effect of late endosomal pH). An insignificant change in the particle size of developed DTsiANp was observed following their incubation in physiological pH 7.4 inferring their capability to remain stable under physiological stability. Upon incubation of DTsiANp at pH 5.5 and pH 4.5, a significant and progressive enhancement in their particle size was observed. The increase in particle size of DTsiANp is ascribed to the presence of dendrimer in the architect of nanoplex that elicits pH-responsive ‘*proton-sponge-effect’*. Our prior research infers the ability of dendrimer to undergo a pH-responsive change in the architecture of dendrimer^40^.

The pH-responsive change in the particle size of DTsiANp was corroborated by an analogous change in the surface zeta potential (*p* < 0.05). In the case of DTsiANp, the surface charge was significantly enhanced to more than 50% at pH 4.5 as compared to their original surface charge at pH 7.4 (Fig. 3D,E). The results conclude the pH-responsive behaviour of DTsiANp owing to the existence of free primary amines in dendrimeric template present in the nanoplex. The amine groups of dendrimers undergo protonation under acidic pH leading to enhancement in net surface positive charge. The protonation of the nanoplex assembly under the acidic environment of endosome generates the repulsive microenvironment in the architect of nanoplex. This repulsive microenvironment existing within the nanoplex leads to an increment in its hydrodynamic crevices volume with a marked increase in ionic concentration osmotically. The cumulative enhancement in size of DTsiANp leads to the swelling of the endosomal compartment ultimately leading to the rupture of the endosomal membrane to mediate endosomal escape of DTsiANp. This event liberates the DTsiANp from the endosomal compartment before its degradation by lysozyme (phenomenon referred as endosomal escape). This outcome is in good agreement with our pH-responsive particle size analysis as discussed previously.

This effect was further confirmed by evaluating the pH-responsive change in the surface zeta potential of plain albumin and dendrimer (Fig. S18). There was no significant effect of pH on the characteristic of siANp due to the absence of dendrimeric template in their architect. Further, it may be noted that the changes in the hydrodynamic particle size and zeta potential of DTsiANp as compared to siANp was not due to the siRNA but was predominantly due to the dendrimeric template. This is because the pH has no notable impact on siRNA and vice versa. The observed effect can be ascribed to the protonation behaviour of dendrimeric template present in the nanoplex^41^. Further, DTsiANp showed the localization in the endo/lysosomal compartment in 8 hr as confirmed by yellow fluorescence of co-localized FAM-siRNA and lyo-tracker dye (Fig. S19). After 8 hr, the diffused red fluorescence suggests the escape of DTsiANp from endo/lysosomal compartment which is evidenced via green fluorescence of FAM-siRNA in the cytoplasmic compartment of the cell. The result ascribed that the existence of dendrimeric template in the DTsiANp provided the endosomal escape capability due to proton sponge effect at the endo/lysozymal pH. The prepared nanoplex were found biocompatible when tested on podocytes and as well as on RBCs inferring them to be safe for *in vivo* administration (Fig. 4G–H; *p* > 0.05). The siRNA bears negative charge and hence poses its interaction as well as permeation across the negatively charged plasma membrane^5,8^. The cationized albumin nanoplex bear the capability to undergo endocytosis^41^. In agreement with this property, the cellular uptake of DTsiANp nanoplex shows 4.45-fold higher cellular uptake as compared to naked siRNA (Fig. 5A). Albumin binds to hormone, transferrin, fatty acid and other hydrophobic molecules in serum^42^. The commercial albumin employed for the preparation of nanoplex comes with ~96% purity, may containing fatty contaminants. It is envisaged that the lipidic fractions fatty acid contaminants present in albumin might be responsible for the passive cellular uptake of siANp (mean fluorescence intensity: 14.38 ± 2.89) via lipidic cell membrane fusion mechanism. Further, it is advocated that the effect of lipidic fractions fatty acid contaminants on membrane fusion of albumin and albumin-based architectures needs to be explored to reach a statistically meaningful conclusion.

Pathologically, in diabetic (HG) condition, the podocytes show hemodynamic and metabolite changes that result in glomerular lesions and proteinuria^31^. HDAC4 is majorly a central molecule that governs the function regulation in podocytes and is also vital to maintain the integrity of podocytes^33^. It was well reported that in HG condition, the overexpression of HDAC4 induces the inflammation, apoptosis, and autophagy in podocytes. Under HG state, HDAC4 gene primarily contributed to podocytes injury in DN^33^. HG exposed podocytes treated with DTsiANp/HDAC4 showed significantly downregulated HDAC4 mRNA expression (27.47 ± 0.27%; *p* < 0.05) (Fig. 5C). This effect can be ascribed to the efficient delivery of HDAC4 siRNA to the podocytes by DTsiANp as compared to DTsiANp/scramble and naked HDAC4 siRNA control. The effect of gene silencing on HDAC4 protein was further confirmed by western blot analysis.

In agreement with the qRT-PCR outcomes, the expression of HDAC4 protein was found to be significantly suppressed following the treatment of DTsiANp/HDAC4 nanoplex by more than 44.024 ± 2.10% (*p* < 0.01), and 45.56 ± 3.61% (*p* < 0.001) as compared to the naked siRNA and DTsiANp/Scramble treated group *in vitro* (Fig. 5D; *p* < 0.05). The treatment of DTsiANp/HDAC4 in C57BL/6 DN mice model significantly lowered the levels of proteinuria (UAER:1.07 ± 0.32) as compared to DTsiANp/scramble and naked siRNA. It is well reported that the knockdown the overexpressed HDAC4 could able to regulate HG-induced transcriptional activity or deacetylation of STAT1 in podocytes leads to suppress inflammation and apoptosis^29^. The effective reduction in UAER with DTsiANp/ HDAC4 infers the superior therapeutic potential of the developed nanoplex (Fig. 6C).

After the treatment of DTsiANp/ HDAC4, the proportion of damaged glomerulus significantly reduced in the kidneys of C57BL/6 DN mice as observed during the renal histopathological analysis Fig. 6F. Upon treatment of DTsiANp/HDAC4, the glomerular area ratio was reduced by almost 0.32-fold as compared to DTsiANp/scramble treated group. As shown in Fig. 6G, the mean glomerular area was reduced after 4-week treatment of HDAC4 siRNA nanoplex indicating the reduction in glomerular injury and ameliorate the podocytes in DN.

In conclusion, the study provides a novel and simple approach to encapsulate, stabilize and deliver loaded siRNA payload with high efficiency. The concept was established and tested using HDAC4 siRNA, however, the same approach can be extended to diverse gene therapeutics including micro RNA (miRNA), oligonucleotide, DNA, etc. A detailed investigation pertains to safety, scalability, and versatility is currently under exploration in our lab. It is expected that the knowledge reported in the work will significantly assist in research aiming at the clinical translation of siRNA therapeutics, which is one of the unmet dire need of a clinician and pharmaceutical industry working towards transforming siRNA as a therapeutic modality. Other innovative versions of this strategy including kidney directed nanoplex is also under exploration and it is expected that in near future more research will come in this line to assist development of a more fitting and clinically translatable generally regarded as safe (GRAS) tag bearing nanoplex for the safe delivery of siRNA and other RNAi gene therapeutics.

## Material and Methods

### Materials

Bovine Serum Albumin-Fraction V was purchased from HiMedia Laboratories GmbH, Germany). Sodium phosphate, sodium chloride, and sodium acetate were purchased from Sigma Aldrich (St. Louis, USA). Scramble (Silencer) 5′-FAM labelled siRNA (sense: 5′-UUCUCCGAACGUGUCACGUdTdT-3′; antisense: 5′-ACGUGACACGUUCGGAGAAdTdT-3′), HDAC4 siRNA (sense: 5′-GGUGCUUAUGGAAAGGGAUTT-3′; antisense: 5′-AUCCCUUUCCAUAAGCACCTT-3′), bicinchoninic acid (BCA) protein assay kit and MTT reagent (3-(4,5-Dimethylthiazol-2-yl)-2,5-Diphenyltetrazolium Bromide) was acquired from Thermo Fisher Scientific (Massachusetts, USA). DEPC treated RNase free water was used for further experimentation with siRNA and procured from Invitrogen (Thermo scientific, Massachusetts, USA). All other reagents and solvents were of analytical grade unless otherwise specified. Agarose and sodium chloride were obtained from Sigma Aldrich (Mumbai, India). RPMI 1640 media, fetal bovine serum (FBS), trypsin-EDTA, penicillin-streptomycin and 1x ITS (Insulin-Transferrin-Selenium) were obtained from Invitrogen and Gibco (Invitrogen, California, USA, Gibco, Life Technologies, Grand Island, USA). Opti-MEM media, Hoechst 33342 were obtained from Gibco, (Life Technologies, Grand Island, USA).

### Cell culture: cell propagation and differentiation

An immortalized human podocyte cell line was gifted by Dr. Jeffrey Kopp, National in the statute of Health (NIH), Maryland, USA, and the cells were cultured as described earlier^43,44^. In brief, podocytes cells were cultured in RPMI 1640 media comprising 10%v/v FBS, 1x ITS (Insulin-Transferrin-Selenium) and 100 IU/ml penicillin and 100 μg/ml streptomycin sulfate as antibiotics. And cells were grown at 33 ± 0.5 °C under 5% CO_2_ condition in Type 1 collagen-coated culture tissue culture flask (25 cm^3^ BD Falcon, Bedford, USA). After 70–80% confluence, cells were shifted to 37 ± 0.5 °C with 5% CO_2_ for 10–14 days for differentiation. High glucose 20–40 mM (30 mM) condition stimulates the expression of HDAC4 in kidney podocytes^33^. Therefore, *in-vitro* DN model i.e. high glucose model was generated on differentiated podocytes cells (6 well plates; 20 × 10^4^ cells/well) using 30 mM glucose containing RPMI 1640 with 1%v/v FBS treated for 48 hr^45,46^. Developed *in vitro* DN model of podocytes were used for different assays as mentioned in respective sections.

### Screening of *d*:siR complex

Dendrimer/siRNA (*d*:siR) complex was prepared by self-assembly method through charged based electrostatic interaction using gentle vortexing^47^. Here, the *d*:siR complexes with different molar ratio were 1:1, 1:2, 1:4 and 1:8 ratio (~1, 0.5, 0.25, and 0.125 *n/p* ratio; (*n*: cationic primary amine groups on polymer, *p*: anionic phosphate groups present on siRNA)). For that, siRNA (200 pmol, 10 µM; 0.266–0.4 µg/µL) was diluted in DEPC treated nuclease-free water and mixed with dendrimer at increasing molecular ratio. For ratios, siRNA and dendrimers were mixed and incubated at room temperature using frequent gentle vertexing (after every 15 min for the 30 s), aid in complex formation. The *d*:siR complex confirmation was done by gel retardation assay and for the surface charge using Malvern zeta sizer ZS-90 (Malvern Instruments, Worcestershire, UK) comprised with He-Ne laser (wavelength: 633 nm).

### Gel retardation assay for determination of *d:*siR ratio

The gel retardation assay was performed to determine *d:*siR ratio and evaluate the progression of dendrimer-siRNA complex formation. The prepared ratios were mixed with 5 µL of 6X loading dye and volume of the samples were adjusted by nuclease-free water. Accurately, 15 µL total sample (260 ng/well siRNA) volume was load into 2% w/v agarose gel comprising ethidium bromide (2 μg/ml) for siRNA visualization. Gel electrophoresis was done in 1X TBE buffer at 80 V and run for 60 min. The retardation of siRNA inside the dendrimer was imaged using UV transilluminator (Bio-rad, USA)^48^.

### Quality-by-Design (QbD) driven optimization of nanoplex preparation methodology

Albumin nanoplex were prepared using a modified protocol of our laboratory to attain nanoplex of target size (≤70 nm), which as per existing reports bear the capability to reach and localize in the podocytes foot process in DN complications^49^. In brief, plain albumin nanoplex (ANp) were prepared via one-step desolvation technique^29,31^. The utilized albumin purity was initially assessed for presence of any other component from the fraction V albumin using matrix-assisted laser desorption/ionization mass spectrophotometry (MALDI-TOF/MS) and sodium dodecyl sulfate-polyacrylamide gel electrophoresis (SDS-PAGE) (*refer supplementary material*). To prepare ANp, a double quantity of ethanol was added dropwise (1 mL/min) in prepared albumin solution (4%w/v; 1 mL) and left under the stirring condition for 2 hr at 1000 rpm (IKA Magnetic Stirrer (RT 5) Germany). Then, the free amino group of albumin nanoplex was cross-linked with genipin (1%w/v) solution and solution was kept for stirring for a further 2 hr at 700 rpm. The nanoplex preparation was accomplished after purification by centrifugation method at 14,000 g for 25 min. The obtained pellet was resuspended in DEPC treated nuclease-free water up to its original volume. To obtain desired particle size (≤70 nm) of ANp, Quality by design approach was applied as discussed in the supplementary section (*refer supplementary material*).

According to the requirement of the experiment, siRNA (200 pmol; approximately 2.5–3.5 µg/µL in DEPC treated nuclease-free water) loaded inside ANp for siANp. Selected *d*:siR (~0.5 *n/p*) ratio was incubated with (4%w/v; 1 mL) albumin for electrostatic interaction for 2 h at 1000 rpm at (37 ± 0.5 °C) and the solution was desolvated using ethanol to obtain DTsiANp/HDAC4. Here, stoichiometric ratio for nanoplex preparation was 1000:0.066:1::Albumin:siRNA:dendrimer. The physicochemical evaluation was done for particle size, surface zeta potential and polydispersity index (PDI) using Zetasizer Nano ZS 90 (Malvern Instruments, UK). Thereafter, nanoplex were lyophilized for stability concern using 3%w/v trehalose^50^. Further DEPC treated nuclease-free PBS (1×) was added to the nanoplex for making the final dose and was utilized for administration.

## Characterization of siRNA Nanoplex

### Dynamic light scattering (DLS), SEM, TEM, and AFM

The prepared and optimized nanoplexs were determined for particle size, polydispersity index (PDI), and zeta potential with the help of Zetasizer (Nano-ZS90, Malvern Instruments, Worcestershire, UK). In brief, suspension of nanoplex was diluted using ultra-pure water and the sample was measured at a fixed angle (173° backscattering). By means of the electrophoretic analyzer, the zeta potential of the nanoplex was measured using Zetasizer (Nano-ZS90, Malvern Instruments, Worcestershire, UK). All the samples were measured at 25 ± 2 °C and the measurements were done in triplicate for evaluation of the efficacy of the results.

Particle size and morphology of prepared nanoplex were determined after lyophilization by means of scanning electron microscopy (SEM) using a JSM-7001FA microscope (JEOL, Tokyo, Japan). Aqueous suspension of nanoplex was kept on a silicon wafer which adheres to a metal stub. Thereafter, mater wafer was dried under vacuum and enclosed with a 20-nm layer of gold. The stubs were observed at an emission of 5.0 kV with 9.5–10.5 mm of working distance^51^. Further, the morphology of DTsiANp was performed through a transmission electron microscope (TEM; Philips, Tecnai 20, Holland; acceleration voltage: 200 kV; magnification: 40,000×). The size of the DTsiANp was measured using AnalySIS software (Soft Imaging Systems, Reutlingen, Germany). A drop of diluted nanoplex was kept on a carbon-coated copper grid. The sample was stained with 1%v/v aqueous solution of phosphotungstic acid and set aside to absorb. After drying, the sample was focused and images were taken^52^. Further, the atomic force microscopy (Probe: SCANASYST-AIR (Modulus range:  <20 MPa; k~ 0.4 N/m nominal, tip radius  <10 nm typical), AFM; Bruker Multimode 8, Bruker, USA) was also performed for DTsiANp^53^. After characterization of nanoplex, the presence of intact albumin in prepared ANp, DTANp, siANp, and DTsiANp was evaluated by means of SDS-PAGE and BCA reagent assay kit. The dendrimeric template was further confirmed in the nanoplex by TNBSA assay, and the changes in surface charge via zeta potential are represented in supplementary file.

### Determination of siRNA encapsulation efficiency of siRNA nanoplex by Gel retardation assay

siRNA binding and encapsulation efficiency of *d*:siR ratio with siANp and DTsiANp were determined using agarose gel electrophoresis. Briefly, naked siRNA was taken as a control and the unbound siRNA from siANp and DTsiANp was separated by means of VivaSpin (MW cut-off: 50 kDa; GE Healthcare, Thermo Scientific, USA) at 16,000 g for 10 minutes at 4 °C. The supernatant was collected and the pellet was redispersed in DEPC treated nuclease-free water (Ambion, USA). Then, nanoplex suspension and supernatant (10 µL) were mixed with the 5 µL (6X) loading dye and made a final volume of 15 µL using DEPC treated nuclease-free water (Ambion, USA). The prepared composition was loaded on to 2%w/v agarose gel and run through Tris-borate (TBE) (40 mM Tris-HCl, 1%v/v acetic acid, 1 mM EDTA) buffer at 80 V. The electrophoretic mobility was analysed by ethidium bromide stained gel using an ultraviolet (UV) illuminator (GelDoc, Bio-Rad, USA)^2^. Further, the actual encapsulation of siRNA inside the nanoplex was confirmed using siRNA protection assay. The experimental protocol as stated in the supplementary material.

### Determination of siRNA entrapment efficiency of siRNA nanoplex by Ribogreen assay

Further, confirmation of encapsulation of siRNA was determined by the amount of extracted siRNA from the lyophilized DTsiANp and siANp as protocol reported by Cun *et al*. with slight modification^54^. For the assay, accurately weighed (1.5 mg) lyophilized nanoplexes was solubilized in 150 µL of chloroform with 500 µL of TE buffer. To extract the siRNA from organic to aqueous phase, the mixture was mixed and rotated continuously at 25 ± 2 °C for about 90 min. Then the mixture was centrifuged at 16,000 g for 25 min at 4 °C to separate an aqueous and organic phase. And the residues of chloroform was removed via incubating the supernatant at 37 ± 2 °C for 5 min. Than the supernatants were diluted with the TE buffer and concentration of the siRNA was measured by means of Ribogreen reagent (Thermo Scientific, USA) based on manufacturer’s instruction via multimode plate reader (excitation wavelength: 485 nm and emission wavelength: 520 nm; Varioskan LUX Multimode, Thermo Fisher Scientific, Massachusetts, USA). Analysis of all samples was performed in triplicate. The encapsulation of siRNA was calculated using actual weight of siRNA (ng) in nanoplex upon the whole weight of nanoplex (mg). As well as, the encapsulation of siRNA in nanoplex was also calculated via the given equation as following:

1 $$siRNA\,encapsulation=\frac{loading\,of\,siRNA}{theoratical\,loading\,of\,siRNA}$$

### Serum stability of siRNA nanoplex

siRNA was found unstable in serum due to the serum nucleases, therefore, after entrapment of *d:*siR ratio, the stability of siRNA in presence of serum was checked for both siANp and DTsiANp with reference to naked siRNA as stated protocol by Tarantula *et al*. with slight modification^55^. For the assay, naked siRNA (260 ng/well siRNA; 10 µM), 1.5 mg (equivalent to 260 ng/well siRNA) siANp and DTsiANp were taken for the investigation. Nanoplexs and naked siRNA were incubated with an equal volume (50 µL) of RPMI 1640 comprised 10%v/v fetal bovine serum (FBS) at 37 ± 0.5 °C. At defined time interval i.e. 0, 1, 2, 4, 6 and 24 hr, aliquots were centrifuged at 21,000 g for 15 min at 4 °C. The supernatant was removed and pallet stored at −20 °C till gel electrophoresis was executed. After 24 hr of sample collection, the stability of siRNA was assessed by gel electrophoresis. For that, aliquots (10 µL) were mixed with the 5 µL (6X) loading dye and prepared composition was loaded on to 2%w/v agarose gel and run through 1 X TBE (Tris-borate) (40 mM Tris-HCl, 1% v/v acetic acid, 1 mM EDTA) buffer at 80 V. The electrophoretic mobility was analyzed due to the ethidium bromide using an ultraviolet (UV) illuminator (GelDoc, Bio-Rad, USA).

Furthermore, the effect of serum on particle size, PDI and surface zeta potential was also evaluated for that siAN and DTsiANp were incubated with an equal volume of RPMI 1640 comprised 10%v/v serum concentration at 37 ± 0.5 °C. At predetermined time point 0, 1, 2, 4, 6 and 24 hr, particle size, PDI and surface zeta potential was measured using Zetasizer Nano ZS90 (Malvern Instruments, UK). All the experiments were performed in triplicate at 37 ± 0.5 °C^51^.

### Endo/Lysosomal Escape tendency of developed siRNA Nanoplex

The amino group presented on the terminal end of the dendrimer was sensitive to endosomal pH (acidic)^41^. Therefore, to understand the effect of environmental pH (physiological and endosomal pH) on the siANp and DTsiANp were treated with phosphate buffer saline (pH 7.4), sodium acetate buffer (pH 5; endosomal pH) and acetate buffer (pH 4.5; late endosomal pH) for assessment of stability. The protocol was followed the same as reported by our group earlier with slight amendments^41^. Optimized ANp and DTANp were incubated with the varied buffer for 0, 1, 2, 4, 6 and 24 hr. The particle size, PDI and zeta potential of the nanoplexs was checked at predetermined time points using Zetasizer Nano ZS90 (Malvern Instruments, UK) to evaluate the stability of the nanoplexs. The effect of microenvironmental pH on plain albumin and dendrimer was also evaluated in terms of zeta potential at different time interval via Zetasizer Nano ZS90. All the experiments were performed in triplicate at 25 ± 2 °C. Further, endo/lysosomal escape activity of nanoplex was evaluated with help of lyso-tracker red dye on HG treated podocytes and protocol was as discussed in the supplementary section (*refer supplementary material*).

### Cell viability assay to elucidate biocompatibility of developed siRNA nanoplex

Initially human kidney podocytes were for cell propagation in 25 cm^3^ T-flask in RPMI 1640 complete medium (contained 10%v/v FBS, 0.5%v/v Insulin-Transferrin-Selenium (ITS) and 1%w/v penicillin-streptomycin antibiotic mixture) at 33 ± 0.5 °C with 5% CO_2_ till 60–80% confluence_._ After that, cells were shifted to 37 ± 0.5 °C with 5% CO_2_ for differentiation for 10–14 days. Fresh medium was provided to cells for three times a week^56,57^. These differentiated cells were utilized for each experiment. After differentiation, human podocytes cells were seeded in 96 well plates (1 × 10^4^ cells/well) and permit to grow overnight in complete RPMI 1640 medium at 37 ± 0.5 °C with 5% CO_2_. Then, cells were treated with HG (30 mM) to induce *in vitro* DN model for 48 hr. After that, the equivalent amount of ANp, DTANp, siANp, and DTsiANp took for treatment (0.625 mg; 1 pmol siRNA/well and incubated for 24 hr. Afterward, MTT (3-(4,5-Dimethylthiazol-2-yl)-2,5-Diphenyltetrazolium Bromide) reagent was added to the cells (20 µL; 5 mg/ml) and incubated for further 4 hr at 37 ± 0.5 °C with 5% CO_2_^58^. MTT solution was replaced with 100 µL DMSO to dissolve the formazan crystals. Untreated cells were taken as control with 100% cell viability. The absorbance was measured at 575 nm using UV microplate reader (Multiscan GO, Thermo Fisher Scientific, Massachusetts, USA) at 37 ± 0.5 °C. The viability of cells was calculated as follows^59^.

2 $$Cell\,viability( \% )=\frac{Abs\,(sample)}{Abs\,(control)}\times 100 \% $$

### Cellular uptake of siRNA nanoplex in HG podocyte DN model

To evaluate the internalization, cellular uptake assay was performed on HG treated podocytes as protocol reported by Huang *et al*. with slight modification^60^. Differentiated podocytes cells were seeded into 6-well plate (2 × 10^5^ cells/well) with a glass coverslip. Cells were treated with high glucose (30 mM; HG)^31^ for 48 hr in presence of serum compromised RPMI 1640 media (1%v/v FBS)^61^ to generate *in vitro* DN model. Media was replaced with Opti-MEM comprised FAM-siRNA loaded DTsiANp (30 pmol of siRNA/well; 10 µM). After 12 hr cellular distribution of siRNA was observed using confocal microscopy (Excitation max: 494 nm; Emission max: 520 nm, Leica Microsystems, Wetzlar, Germany)^60^.

### Gene silencing efficiency: Quantitative RT-PCR

Real-time PCR was executed to evaluate the HDAC4 silencing efficiency of HDAC4 loaded nanoplex. Differentiated podocytes cells were grown in 6 well plates (2 × 10^5^ cells/ well) in RPMI 1640 complete medium till 60% confluence achieved. Then, media was removed and incubated with HG (30 mM) comprised serum compromised RPMI 1640 media (1%v/v FBS) for 48 hr^61^. Afterward, media was replenished with Opti-MEM containing DTsiANp/ HDAC4 (~30pmol siRNA/well) and incubated for 24 hr. Here, DTsiANp/Scramble selected as a negative control. After treatment, total RNA was extracted using RNeasy mini kit (Qiagen, Hilden, Germany) as stated by manufacturer’s protocol and quantified using Nanodrop-2000 spectrophotometer (Thermo Fisher Scientific, Massachusetts, USA). The cDNA was prepared by iscript cDNA synthesis kit (Bio-Rad, California, USA). Then, HDAC4 gene expression was quantified with a 1:10 dilution of cDNA using the iScript SYBR green supermix (Bio-Rad, California, USA) through StepOne Real-time PCR, (Applied Biosystems, California, USA). PCR KiCqStart primers were utilized to amplify 18 s (forward: 5′-GTAACCCGTTGAACCCCATT-3′ and reverse: 5′-CCATCCAATCGGTAGTAGCG-3′) and HDAC4 gene (forward: 5′-AGTGTCGACCTCCTATAACCA-3′ and reverse: 5′-GCTTTAGCCTGGACCGTAAT-3′). The HDAC4 expression level was analyzed via Ct values (cycle threshold). The experiment was performed in triplicate^62^.

### HDAC4 expression by western blot analysis: *In vitro* and *in vivo*

To evaluate the expression of HDAC4 in podocytes cells and mouse model western blot was performed. After differentiation podocytes, cells were seeded in 6 well plates (2 × 10^5^ cells/well) in RPMI 1640 complete medium and treated with HG (30 mM) in serum compromised media (RPMI 1640 media with 1%v/v FBS) for 48 hr^31^. DTsiANp/ HDAC4 (30 pmol/well siRNA; 6.5 mg nanoplex) and DTsiANp/ Scramble was treated for 48 hr. The protein was extracted and lysate resuspended in RIPA lysis buffer comprising 1 µL of protease inhibitor cocktail. Protein concentration from podocytes cell and tissue was estimated using BCA reagent. Protein (25 µg) was separated via SDS-PAGE (acrylamide gel 10%) and transferred over PVDF membrane using RTA Trans Turbo kit (Bio-Rad, USA). Then, specific protein detection was done using incubation with primary antibody against (HDAC4 1:1000, Abcam, Cambridge, UK) and (β-actin 1:5000, Santacruz, USA) at 4 °C overnight. After completion of incubation with primary antibody, membrane washed with 0.1% TBST following that incubation with HRP-conjugated secondary antibody ((Goat anti-mouse IgG-HRP 1:20000, Santacruz and Goat anti-rabbit IgG-HRP 1:20000, Abcam) at room temperature for 2 hr. Detection of bands were using chemiluminescence substrate (Bio-Rad, USA)). Furthermore, Band intensity was quantified by means of ImageJ software (NIH, Bethesda, MD).

For *in vivo* western blot analysis healthy mice kidney, STZ induced DN control mice and nanoplex treated kidney was homogenate using tissue lyser (Tissue Lyser LT, Qiagen, Germany) and centrifuged (12,000 g for 5 min) for remaining of tissue removal. The collected supernatant was utilized for protein extraction and protein content measurement using BCA reagent and western blots analysis was performed as mentioned procedure above.

### Haemocompatibility assay

To evaluate the biocompatibility of nanoplex haemocompatibility assay was performed^41,51^. Briefly, rat blood was taken in heparinized vials (5000 I.U./mL, Himedia, Mumbai, India) centrifuged at 1000 g for 10 min for RBCs pallet. The pellet was washed with the 0.9%w/v normal saline (5X). A 2%v/v RBC solution was prepared for experimentation. RBC solution was incubated with ANp, DTANp (1, 2.5 and 5 µg/ml concentrations of dendrimer), saline (0% hemolysis; negative control), and Triton X-100 (0.1%v/v) (100% hemolysis; positive control) for 2 hr at 37 ± 0.5 °C to allow interaction. After 2 hr, the suspension was centrifuged at 1000 g for 10 min and the supernatant obtained in each group was analyzed for the hemoglobin content via absorbance at 540 nm using UV microplate reader (Varioskan LUX Multimode, Thermo Fisher Scientific, Massachusetts, USA). The percentage of hemolysis was determined by using the following equation:

3 $$ \% Hemolysis=100\times \frac{(Abs.Sample-Abs.Negative\,Control)}{(Abs.Positive\,Control-Abs.Negative\,Ccontrol)}$$

where Abs. Sample detonated for Absorbance of the sample, Absorbance of the negative control (0.9%w/v saline), Absorbance of positive control (Triton-X).

### Experimental Animal model and treatment

All animal studies were performed in accordance with guidelines and protocols with National Institute of pharmaceutical education and research (NIPER) Guidelines for Care and Use of Laboratory Animals. All procedures and protocols followed in this study were approved by Institutional Animal Ethics Committee (IAEC) at NIPER-Ahmedabad, Gujarat, India vide approval letter number: NIPER-A/IAEC/2017/034. C57BL/6 male mice (average body weight: 18–23 g) (Supplier: Zydus Research Centre, Ahmedabad) were injected with 50 mg/kg of streptozotocin (STZ) (0.1 M citrate buffer (pH 4.5) intraperitoneally on 5 consecutive days to circumvent acute toxicity of STZ. Control animal was received citrate buffer only. Confirmation of diabetes was done through tail vein blood glucose levels (fasting glucose> 12 mmol; Accu Chek-Active glucometer, Roche, USA). Mice were divided randomly into an experimental group (per group six mice). All mice were sacrificed at 4 weeks post-injection of naked HDAC4 siRNA, DTsiANp/scramble, DTsiANp/HDAC4 to diabetes mice. The developed siRNA nanoplex was given to mouse via tail vein at 1 mg/kg dose for twice a week. After treatment, from the freshly harvested kidney glomerular portion was isolated from half kidney and homogenate the using tissue lyser (TissueLyser LT, Qiagen, Germany) then centrifuged (12,000 g for 5 min) to remove tissue remains. The supernatant was collected for protein extraction for Western blots analysis^63^. Other half portion was fixed in formalin for histological evaluation of glomeruli. Later completion of treatment, urinary albumin concentrations were measured with the Albumin mouse ELISA kit (ab108792, Abcam, Cambridge, UK) and represented as urine albumin excretion ratio (UAER)^64^.

### Renal histopathological analysis

Isolated kidney tissue was taken for histological evaluation, fixed in 4% paraformaldehyde and embedded in paraffin. 5-µm thick sections were processed for hematoxylin and eosin staining according to manufacturer’s protocol. Morphological evaluation and semi-quantitative analysis of section were done for the glomerular area for glomerular injury using ImageJ software. The glomerular area was determined via ImageJ software using 15 random cortex region image per mouse under a low magnification field of vision (400×)^65^.

### Statistical analysis

Results are represented as mean ± S.D or mean ± SEM. Statistical differences among the groups were assessed by means of one-way analysis of variance (ANOVA) for *in vitro* and *in vivo* data analysis. Following that Bonferroni’s post-test was applied to all pair of columns with control as well. *p* < 0.05 was considered significant. Statistical analysis was done through Graph Pad Prism (GraphPad software, SPSS, Chicago, IL, USA).

## Supplementary information

Supplimentary File

## Data Availability

The author’s consent to make materials, data and associated protocols promptly available to readers without undue qualifications in material transfer agreements.

## References

[1] Setten, R. L., Rossi, J. J. & Han, S.-P. The current state and future directions of RNAi-based therapeutics. *Nature Reviews Drug Discovery*, **1** (2019). 10.1038/s41573-019-0017-4 30846871

[2] Youngren, S. R., Tekade, R. K., Gustilo, B., Hoffmann, P. R. & Chougule, M. B. STAT6 siRNA matrix-loaded gelatin nanocarriers: formulation, characterization, and *ex vivo* proof of concept using adenocarcinoma cells. *BioMed research international* **2013** (2013). 10.1155/2013/858946 PMC3806510 24191252

[3] Kumar Tekade R, GS Maheshwari R, Sharma A, Tekade P, Singh Chauhan M (2015). A. siRNA therapy, challenges and underlying perspectives of dendrimer as delivery vector. Current pharmaceutical design.

[4] Zhu J (2018). Dual-responsive polyplexes with enhanced disassembly and endosomal escape for efficient delivery of siRNA. Biomaterials.

[5] Aagaard L, Rossi JJ (2007). RNAi therapeutics: principles, prospects and challenges. Advanced drug delivery reviews.

[6] Almutiri, S., Berry, M., Logan, A. & Ahmed, Z. Non-viral-mediated suppression of AMIGO3 promotes disinhibited NT3-mediated regeneration of spinal cord dorsal column axons. *Scientific reports* **8** (2018). 10.1038/s41598-018-29124-z PMC6048058 30013050

[7] Majowicz A (2017). Successful repeated hepatic gene delivery in mice and non-human primates achieved by sequential administration of AAV5ch and AAV1. Molecular Therapy.

[8] Yin H (2014). Non-viral vectors for gene-based therapy. Nature Reviews Genetics.

[9] Nakamura T (2016). Small-sized, stable lipid nanoparticle for the efficient delivery of siRNA to human immune cell lines. Scientific reports.

[10] Kasuya T (2016). Ribonuclease H1-dependent hepatotoxicity caused by locked nucleic acid-modified gapmer antisense oligonucleotides. Scientific reports.

[11] Kasinski AL (2015). A combinatorial microRNA therapeutics approach to suppressing non-small cell lung cancer. Oncogene.

[12] Nair JK (2014). Multivalent N-acetylgalactosamine-conjugated siRNA localizes in hepatocytes and elicits robust RNAi-mediated gene silencing. Journal of the American Chemical Society.

[13] Orellana EA (2017). FolamiRs: Ligand-targeted, vehicle-free delivery of microRNAs for the treatment of cancer. Science translational medicine.

[14] Whitehead KA (2014). Degradable lipid nanoparticles with predictable *in vivo* siRNA delivery activity. Nature communications.

[15] Morrison, C. Alnylam prepares to land first RNAi drug approval. *Nature Reviews Drug Discovery* **17**, 156–157 (2018). 10.1038/nrd.2018.20 29487392

[16] Adams D (2018). Patisiran, an RNAi therapeutic, for hereditary transthyretin amyloidosis. New England Journal of Medicine.

[17] Kauffman KJ, Webber MJ, Anderson DG (2016). Materials for non-viral intracellular delivery of messenger RNA therapeutics. Journal of Controlled Release.

[18] Villar-Alvarez E (2019). siRNA Silencing by Chemically Modified Biopolymeric Nanovectors. ACS Omega.

[19] Zhao Y (2018). Fine Tuning of Core–Shell Structure of Hyaluronic Acid/Cell-Penetrating Peptides/siRNA Nanoparticles for Enhanced Gene Delivery to Macrophages in Antiatherosclerotic Therapy. Biomacromolecules.

[20] Copolovici DM, Langel K, Eriste E, Langel U (2014). Cell-penetrating peptides: design, synthesis, and applications. ACS nano.

[21] Fakih, H. H., Fakhoury, J. J., Bousmail, D. & Sleiman, H. F. Minimalist Design of a Stimuli-Responsive Spherical Nucleic Acid for Conditional Delivery of Oligonucleotide Therapeutics. *ACS applied materials & interfaces* (2019). 10.1021/acsami.8b18790 30720262

[22] Wu C, Li J, Wang W, Hammond PT (2018). Rationally designed polycationic carriers for potent polymeric siRNA-mediated gene silencing. ACS nano.

[23] Elzoghby AO, Samy WM, Elgindy NA (2012). Albumin-based nanoparticles as potential controlled release drug delivery systems. Journal of controlled release.

[24] Houghton PJ (2015). Initial testing (stage 1) of the tubulin binding agent nanoparticle albumin-bound (nab) paclitaxel (Abraxane®) by the Pediatric Preclinical Testing Program (PPTP). Pediatric blood & cancer.

[25] Wu L (2017). Albumin-based nanoparticles as methylprednisolone carriers for targeted delivery towards the neonatal Fc receptor in glomerular podocytes. International journal of molecular medicine.

[26] Kratz F (2008). Albumin as a drug carrier: design of prodrugs, drug conjugates and nanoparticles. J Control Release.

[27] Wen H, Yin Y, Huang C, Pan W, Liang D (2017). Encapsulation of RNA by negatively charged human serum albumin via physical interactions. *Science China*. Chemistry.

[28] Hadden M, Advani A (2018). Histone deacetylase inhibitors and diabetic kidney disease. International journal of molecular sciences.

[29] Wei Q, Dong Z (2014). HDAC4 blocks autophagy to trigger podocyte injury: non-epigenetic action in diabetic nephropathy. Kidney international.

[30] Singh HD, Wang G, Uludağ H, Unsworth LD (2010). Poly-L-lysine-coated albumin nanoparticles: stability, mechanism for increasing *in vitro* enzymatic resilience, and siRNA release characteristics. Acta biomaterialia.

[31] Zhou Z (2017). MicroRNA-27a promotes podocyte injury via PPARγ-mediated β-catenin activation in diabetic nephropathy. Cell death & disease.

[32] Huang G (2017). Notoginsenoside R1 attenuates glucose-induced podocyte injury via the inhibition of apoptosis and the activation of autophagy through the PI3K/Akt/mTOR signaling pathway. International journal of molecular medicine.

[33] Wang X (2014). Histone deacetylase 4 selectively contributes to podocyte injury in diabetic nephropathy. Kidney international.

[34] Am Hong C, Son HY, Nam YS (2018). Layer-by-layer siRNA/poly (L-lysine) Multilayers on Polydopamine-coated Surface for Efficient Cell Adhesion and Gene Silencing. Scientific reports.

[35] Monnery BD (2017). Cytotoxicity of polycations: relationship of molecular weight and the hydrolytic theory of the mechanism of toxicity. International journal of pharmaceutics.

[36] Du, B., Yu, M. & Zheng, J. Transport and interactions of nanoparticles in the kidneys. *Nature Reviews Materials*, **1** (2018).

[37] Chen D (2018). Kidney-targeted drug delivery via rhein-loaded polyethyleneglycol-co-polycaprolactone-co-polyethylenimine nanoparticles for diabetic nephropathy therapy. International journal of nanomedicine.

[38] Tan YL, Ho HK (2018). Navigating albumin-based nanoparticles through various drug delivery routes. Drug discovery today.

[39] Wu Z (2018). Tumor Microenvironment-Response Calcium Phosphate Hybrid Nanoparticles Enhanced siRNAs Targeting Tumors *In Vivo*. Journal of biomedical nanotechnology.

[40] Tekade, R. K. & Chougule, M. B. Formulation development and evaluation of hybrid nanocarrier for cancer therapy: Taguchi orthogonal array based design. *BioMed research international* **2013** (2013). 10.1155/2013/712678 PMC3784087 24106715

[41] Tekade RK, Tekade M, Kumar M, Chauhan AS (2015). Dendrimer-stabilized smart-nanoparticle (DSSN) platform for targeted delivery of hydrophobic antitumor therapeutics. Pharmaceutical research.

[42] Coverdale, J. P., Khazaipoul, S., Arya, S., Stewart, A. J. & Blindauer, C. A. Crosstalk between zinc and free fatty acids in plasma. *Biochimica et Biophysica Acta (BBA)-Molecular and Cell Biology of Lipids* (2018). 10.1016/j.bbalip.2018.09.007 PMC6372834 30266430

[43] Kopp, J. B. & Heymann, J. c-Src is in the effector pathway linking uPAR and podocyte injury. *The Journal of clinical investigation* **129** (2019). 10.1172/JCI127927 PMC6486326 30939121

[44] Saleem MA (2002). A conditionally immortalized human podocyte cell line demonstrating nephrin and podocin expression. Journal of the American Society of Nephrology.

[45] Liu B-C (2013). High glucose induces podocyte apoptosis by stimulating TRPC6 via elevation of reactive oxygen species. Biochimica et Biophysica Acta (BBA)-Molecular Cell Research.

[46] Imasawa T (2016). High glucose repatterns human podocyte energy metabolism during differentiation and diabetic nephropathy. The FASEB Journal.

[47] Greco CT, Muir VG, Epps TH, Sullivan MO (2017). Efficient tuning of siRNA dose response by combining mixed polymer nanocarriers with simple kinetic modeling. Acta biomaterialia.

[48] Sarett SM (2016). Hydrophobic interactions between polymeric carrier and palmitic acid-conjugated siRNA improve PEGylated polyplex stability and enhance *in vivo* pharmacokinetics and tumor gene silencing. Biomaterials.

[49] Wang J, Masehi-Lano JJ, Chung EJ (2017). Peptide and antibody ligands for renal targeting: nanomedicine strategies for kidney disease. Biomaterials science.

[50] Wang L (2018). Cryoprotectant choice and analyses of freeze-drying drug suspension of nanoparticles with functional stabilisers. Journal of microencapsulation.

[51] Muniswamy VJ (2019). ‘Dendrimer-Cationized-Albumin’encrusted polymeric nanoparticle improves BBB penetration and anticancer activity of doxorubicin. International journal of pharmaceutics.

[52] Raval N, Khunt D, Misra M (2018). Microemulsion-based delivery of triamcinolone acetonide to posterior segment of eye using chitosan and butter oil as permeation enhancer: an *in vitro* and *in vivo* investigation. Journal of microencapsulation.

[53] Vergaro V (2015). Interaction between human serum albumin and different anatase TiO2 nanoparticles: A nano-bio interface study. Nanomaterials and Nanotechnology.

[54] Cun D (2011). High loading efficiency and sustained release of siRNA encapsulated in PLGA nanoparticles: quality by design optimization and characterization. European Journal of Pharmaceutics and Biopharmaceutics.

[55] Taratula O (2009). Surface-engineered targeted PPI dendrimer for efficient intracellular and intratumoral siRNA delivery. Journal of Controlled Release.

[56] Perche F, Patel NR, Torchilin VP (2012). Accumulation and toxicity of antibody-targeted doxorubicin-loaded PEG–PE micelles in ovarian cancer cell spheroid model. Journal of controlled release.

[57] Shankland S, Pippin J, Reiser J, Mundel P (2007). Podocytes in culture: past, present, and future. Kidney international.

[58] Zhang H, Kong X, Tang Y, Lin W (2016). Hydrogen Sulfide Triggered Charge-Reversal Micelles for Cancer-Targeted Drug Delivery and Imaging. ACS applied materials & interfaces.

[59] Ji S (2012). RGD-conjugated albumin nanoparticles as a novel delivery vehicle in pancreatic cancer therapy. Cancer biology & therapy.

[60] Liu J (2015). Integrin-targeted pH-responsive micelles for enhanced efficiency of anticancer treatment *in vitro* and *in vivo*. Nanoscale.

[61] Oe Y (2014). Actively-targeted polyion complex micelles stabilized by cholesterol and disulfide cross-linking for systemic delivery of siRNA to solid tumors. Biomaterials.

[62] Weber N (2008). Characterization of carbosilane dendrimers as effective carriers of siRNA to HIV-infected lymphocytes. Journal of Controlled Release.

[63] Jha JC (2016). Podocyte-specific Nox4 deletion affords renoprotection in a mouse model of diabetic nephropathy. Diabetologia.

[64] Gai Z (2014). Uninephrectomy augments the effects of high fat diet induced obesity on gene expression in mouse kidney. Biochimica et Biophysica Acta (BBA)-Molecular Basis of Disease.

[65] Grange C (2019). Stem cell-derived extracellular vesicles inhibit and revert fibrosis progression in a mouse model of diabetic nephropathy. Scientific reports.

