# Supplementary material for: Method and its Composition for encapsulation, stabilization, and delivery of siRNA in Anionic polymeric nanoplex: An In vitro- In vivo Assessment
Source: Sci Rep. 2019 Nov 5;9:16047. doi: 10.1038/s41598-019-52390-4 (PMC6831632; doi:10.1038/s41598-019-52390-4)
Supplement: Supplementary file 1 — Supplimentary File [file 41598_2019_52390_MOESM1_ESM.pdf]

Supplementary material

Method and its Composition for encapsulation, stabilization, and delivery of siRNA in Anionic polymeric Nanoplex: An *In vitro- In vivo* Assessment

Nidhi Raval, Hardi Jogi, Piyush Gondaliya, Kiran Kalia, Rakesh K. Tekade*

National Institute of Pharmaceutical Education and Research (NIPER) – Ahmedabad, Palaj, (An Institute of National Importance), Opposite Air Force Station, Gandhinagar-382355, Gujarat, India

***Corresponding Author**

Rakesh K. Tekade, Ph.D

National Institute of Pharmaceutical Education and Research (NIPER) Ahmedabad

(An Institute of National Importance, Government of India)

Department of Pharmaceuticals, Ministry of Chemicals and Fertilizers,

Palaj, Opp. Air force station,

Gandhinagar-382355, Gujarat, India

Office Tel: 079-6674550, 66745555, Fax-07966745560

Email: rakeshtekade@gmail.com

QbD-driven Synthesis and Optimization of nanoplex

Preliminary screening of process variables using Two-level Five-factor (2^5) full factorial design. The parameters for the synthesis of nanoplex was optimized through Design of experiment-driven (DoE) Quality by design approach (QbD) to achieve nanoplex with desired particle size and PDI. For this, first of all, the critically quality attributes (CQA) were selected through existing literature. This CQA was identified to be a concentration of albumin, the concentration of the desolvating agent, its type, rate of addition of desolvating agent, reaction time between the polymer and desolvating agent, etc. As per the lead from available literature that reports the *in vivo* biodistribution of albumin-based nanoplex (ANp), the hydrodynamic particle size, and PDI is the most significant CQA that significantly alters the biodistribution of nanoplex in vital body organs including kidney uptake and retention[1]; and hence the same has been considered for the optimization of nanoplex

Screening of process variables was done by the statistical analysis at Two-level Five-factor (2^5), which was selected by means of preliminary wet-lab experiments by applying Two-level full factorial design. The formula generated by QbD Design-Expert Software (Stat-Ease, Version 7.0; Minneapolis, MN) concerning were prepared in triplicate and the outcomes feed into the software feed section. The screened process variable with their outcomes as stated in **Table S1.**

Supplementary Table S1: Screening of process variables using Two-level Five-factor (2^5) full factorial design

Batch No.	Factors					Results	
	Albumin *(%w/v)*	Ethanol volume *(% v/v)*	Agitation speed *(rpm)*	Ethanol concentration *(% v/v)*	Rate of addition (µl/min)	Particle Size (nm)	PDI
A1	2	200	500	40	60	134.15 ±5.20	0.610 ±0.09
A2	6	200	500	40	20	246.3 ±20.60	0.595 ±0.23
A3	2	600	500	40	20	142.50±15.90	0.126 ±0.08
A4	6	600	500	40	60	250.66±23.68	0.606 ±0.11
A5	2	200	1000	40	20	301.42±19.30	0.630 ±0.18
A6	6	200	1000	40	60	477.23±18.80	0.672 ±0.08
A7	2	600	1000	40	60	400.3 ±20.20	0.620 ±0.10
A8	6	600	1000	40	20	340.17±17.50	0.551 ±0.070
A9	2	200	500	100	20	108.33±10.30	0.320 ±0.00
A10	6	200	500	100	60	86.75 ±2.60	0.455 ±0.06
A11	2	600	500	100	60	60.22 ±1.90	0.082 ±0.01
A12	6	600	500	100	20	150.00 ±6.40	0.480 ±0.01

A13	2	200	1000	100	60	72.05 ±0.86	0.271 ±0.02
A14	6	200	1000	100	20	71.04 ±1.60	0.423 ±0.08
A15	2	600	1000	100	20	63.57 ±0.28	0.181 ±0.02
A16	6	600	1000	100	60	252.23 ±6.80	0.582 ±0.15

Results are represented as mean ±S.D (n=3).

It was found that the contribution of albumin concentration, ethanol concentration and agitation speed towards the resultant particle size was found 8.37 %, 50.47 %, and 16.04 %, respectively as shown in **Fig. S1A**. The *p*-value for the two-level Five-factor (2^5) full factorial design model for particle size was 0.0046 (R^2 value: 0.8989), which indicated that the model was significant for process outcomes. Notably, the *p*-value for albumin concentration (0.0806), ethanol volume (0.6132) and addition rate (0.6132) was larger than 0.05 and hence was found non-significant to the applied model. Therefore, it suggested that albumin concentration, ethanol concentration, and agitation speed has maximum influence on the particle size of nanoplex.

In context to PDI, the contribution of albumin concentration, ethanol volume, and agitation speed (rpm) was found 21.19 %, 32.71 %, and 7.32 %, respectively (**Fig. S1B**). However, *p*-value and R^2 value for the model obtained by applying ANOVA were 0.0254 and 0.9607, respectively signifies that the applied model was significant to process outcomes. On another hand, the *p*-value for ethanol volume (0.3943), rate of addition (0.2888) and agitation speed (0.1623) were found to be greater than 0.05, which infers that it does not significantly affect on the PDI of the resultant nanoplex. Similar to particle size, in the case of PDI, albumin concentration, ethanol concentration and agitation speed have shown the maximum impact on PDI of nanoplex (**Fig. S1B**). Therefore, these process parameters need more optimization to attain the targeted size and PDI.

In summary, taking lad from the QbD-driven risk factor analysis using 2-level full factorial design, ethanol volume and rate of addition were kept constant, since they had no a significant impact on particle size and PDI of nanoplex. The ethanol volume and rate of addition were kept constant at 4 ml and 50 µl/min, respectively. The albumin concentration (%w/v), ethanol concentration (%v/v) and agitation speed (rpm) were selected as CQA for in-depth three-factor at three-level (3^3) Box-Behnken design due to their direct and significant impact on the particle size and PDI of nanoplex. It may be noted that three factors at three-level Box-Behnken design have been selected for QbD optimization due to the selected number of factors.

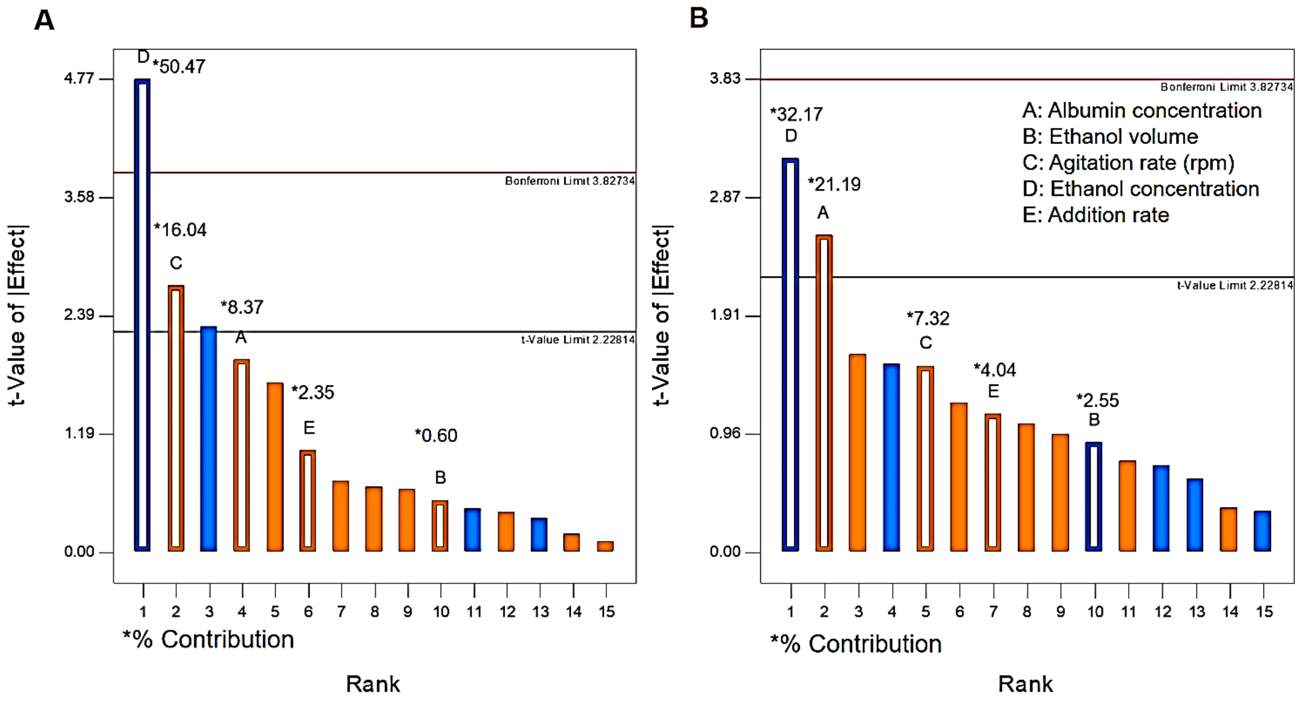

Supplementary Figure S1. Pareto chart showing percentage contribution of selected factors (A) Particle size (B) PDI.

QbD-driven Optimization of screened process parameters using Box-Behnken design (3^3).

The selected process parameters **(Table S2)** were tested by applying Box-Behnken design to assess the ideal process conditions for attaining the nanoplex of the desired size. It may be noted that the particle size of nanoplex governs the kidney uptake and retention of albumin nanoplex [2]. As per the reported literature, the uniform albumin nanoparticle of size ≤ 70 nm selectively shows uptake in the kidney with preferential localization in the kidney podocytes [3]. The outcome of this design confirms the agitation speed, ethanol concentration and albumin concentration of 100 rpm, 1000 %v/v and 4 %w/v, respectively produces the nanoplex of desired particle size 66.23 ±1.29 (Target size: ≤70 nm size), and PDI 0.280 ±0.31 (Target PDI: < 0.3).

Supplementary Table S2: Resultant responses obtained from Box-Behnken design.

Batch	Variables in Box-Behnken design			Predicted Outcome		Experimental Outcome	
	**Albumin concentration (% w/v)**	**Ethanol Concentration (%v/v)**	**Agitation speed (rpm)**	**Particle size (nm)**	**PDI**	**Particle size (nm)**	**PDI**
B1	2	40	750	136.64	0.57	137.83 ±5.30	0.548 ±0.08
B2	6	40	750	146.28	0.65	154.17 ±23.95	0.637 ±0.21
B3	2	100	750	86.29	0.26	75.31 ±5.93	0.279 ±0.06
B4	6	100	750	103.74	0.27	103.56 ±6.22	0.290 ±0.00
B5	2	70	500	106.50	0.27	116.74 ±25.96	0.300 ±0.08
B6	6	70	500	129.21	0.42	129.56 ±22.98	0.439 ±0.25
B7	2	70	1000	76.98	0.43	76.63 ±7.19	0.411 ±0.11
B8	6	70	1000	81.34	0.36	71.10 ±1.32	0.335 ±0.04
B9	4	40	500	152.08	0.56	139.75 ±22.24	0.562 ±0.21
B10	4	100	500	77.86	0.31	79.61 ±1.64	0.265 ±0.07
B11	4	40	1000	85.61	0.70	83.87 ±17.56	0.742 ±0.35
B12	4	100	1000	66.94	0.27	66.23 ±1.29	0.280 ±0.31
B13	4	70	750	76.82	0.50	77.59 ±2.07	0.486 ±0.01
B14	4	70	750	76.82	0.50	77.75 ±0.92	0.445 ±0.02
B15	4	70	750	76.82	0.50	95.03 ±2.82	0.505 ±0.04
B16	4	70	750	76.82	0.50	71.64 ±3.21	0.547 ±0.06
B17	4	70	750	76.82	0.50	62.10 ±0.88	0.504 ±0.03

The experiments were performed on the basis of the runs given by the Design-Expert Software (Stat-Ease, Version 7.0; Minneapolis, MN). Red color coding represents nonacceptable experimental outcome, while green color coding represents acceptable experimental outcomes. Results are represented as mean±S.D (n=3).

Using Box-Behnken design, the polynomial equation for particle size was also obtained as follows to assess the influence of individual process parameters on the process outcome:

Particle Size (Y_1) = 76.82+6.77A-23.22B -19.35C+1.95AB-4.59AC+13.89BC+22.15A^2+19.27B^2-0.47C2……………………………………. Equation (S1)

Here A; Albumin concentration (%w/v), B; ethanol concentration (%v/v) and C; agitation speed (rpm). AB, Interaction between albumin concentration and ethanol concentration; BC, Interaction between ethanol concentration and agitation speed; AC, Interaction between albumin concentration and agitation speed. From Equation S1, it can be concluded that ethanol concentration and agitation speed have a negative impact on the particle size of the nanoplex. This suggests that upon increasing the values of these variables, a net decrement in particle size of nanoplex is obtained. The concentration of albumin showed a positive impact on the particle size

of nanoplex, suggesting that the decrease in the albumin concentration results in a smaller particle size of nanoplex. Furthermore, the interaction between AB and BC had positive interaction while albumin concentration (A) and agitation speed (C) attained negative interaction. Hence, Albumin concentration was found as one of the most critical parameters for attaining nanoplex of desired particle size.

The obtained polynomial equation for the estimation of PDI from the Box-Behnken design was;

$$\text{PDI } (Y_2) = 0.50 + 0.02A - 0.17B + 0.024C - 0.02AB - 0.054AC - 0.044BC - 0.073A^2 + 0.015B^2 - 0.053C^2 \qquad \text{Equation S2}$$

The Equation (S2) suggests that variable B had a negative effect on PDI of the nanoplex. Thus, if increment in the concentration of ethanol smaller size would be attained due to the surface tension of the particles. On another hand, A and C showed a positive influence on the response. Therefore, it suggests that if the concentration of albumin and agitation speed remain optimum than lesser PDI would be attained due to even folding of the polymer. However, in terms of interaction, all other factors displayed negative interaction for PDI.

It may be noted that for a significant correlation among the applied Box-Behnken design model and CQA selected in the experiment, it is advocated that the *p*-value should be $p<0.05$ (nonsignificant difference) and R^2 must be ≈1 as per the QbD guideline[4,5]. Hence, the *p*-value was determined for the applied Box-Behnken design, wherein, the values were found to be 0.0092 and 0.0004, respectively for the particle size and PDI of nanoplex. The R^2 for the set of the experiment was found to be 0.8989 and 0.9607, respectively for the particle size and PDI of nanoplex inferring significant correlation among the applied Box-Behnken design model and CQA selected in the experiment.

Further, a comprehensive evaluation of the inter-relationship among the process variables on the process responses, the 3D-surface response contour plots were obtained employing the Design-Expert Software (Stat-Ease, Version 7.0; Minneapolis, MN) concerning the particle size and PDI as the net endpoint of the process**. Fig. S2A** suggests that at lesser agitation speed (500 rpm) and optimum albumin concentration and at higher ethanol concentration led to smaller the PDI. But at intermediate agitation speed, the obtained desirable region (blue region) was very less compared to higher agitation speed (1000 rpm) (**Fig. S2B-C**). Therefore, intermediate albumin concentration

and high concentration of ethanol, with higher agitation speed led to lesser the PDI as shown in interaction curves of the surface response plot **(Fig. S2C).**

Similarly, the 3D-surface response contour plots inferred that at lesser agitation speed (500 rpm) obtained desirability region was very less (blue region in **Fig. S2D**). Whereas, as shown in **Fig. S2E-F** higher as the agitation speed (750 and 1000 rpm) showed more the desirability area and led to lesser particle size for nanoplex. The highest desirable region was obtained at 1000 rpm agitation speed with optimum concentration of albumin (4 %w/v) and a high concentration of ethanol concentration (100 %v/v). This optimized process formula was further validated for reproducibility.

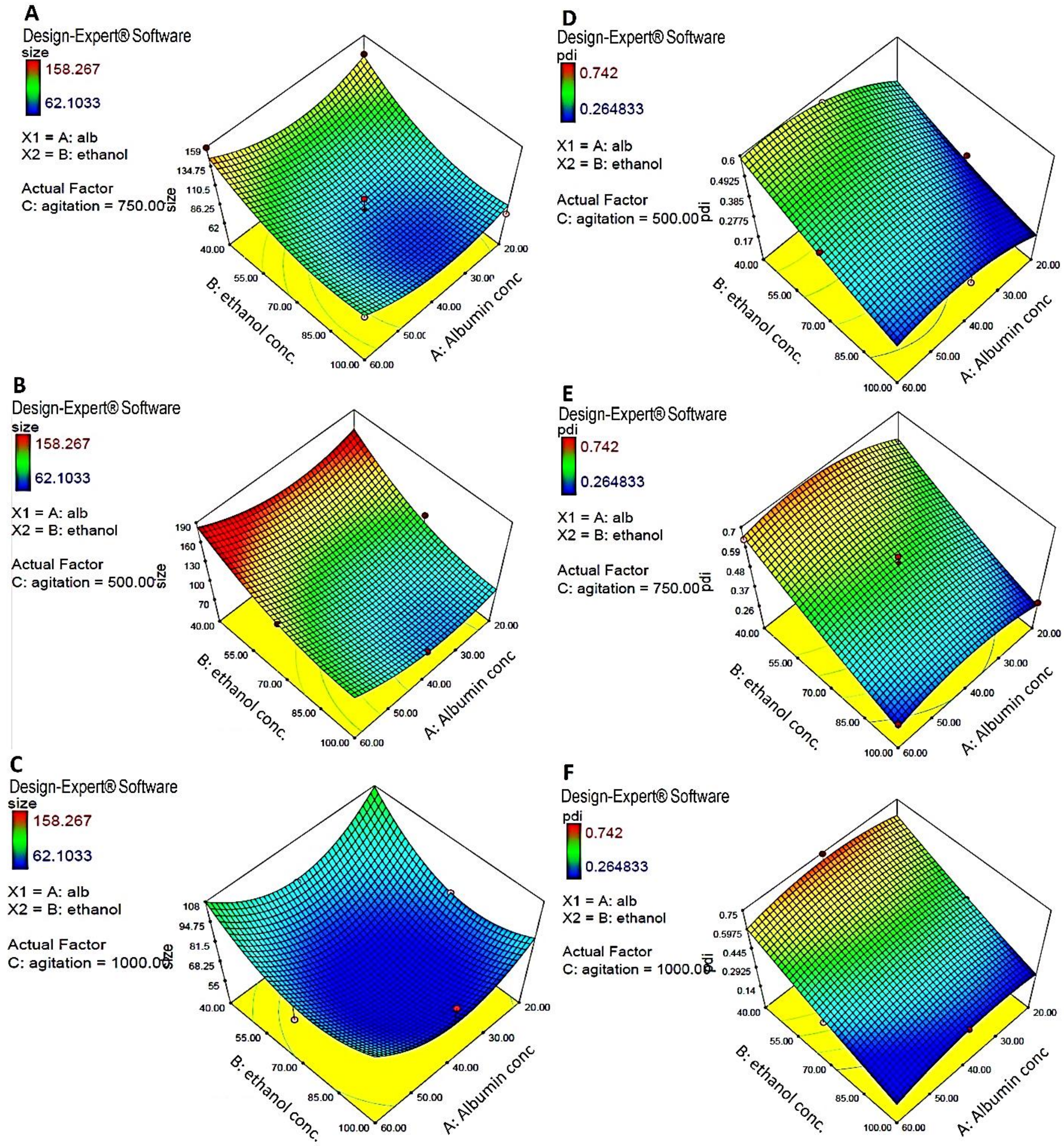

Supplementary Figure S2. 3D surface plot of different agitation speed for particle size (A) contour plot at 500 rpm (B) contour plot at 750 rpm (C) contour plot at 1000 rpm. 3D surface plot of different agitation speed for PDI (D) contour plot at 500 rpm (E) contour plot at 750 rpm (F) contour plot at 1000 rpm.

Validation of QbD Design and Model. The design space was generated with a targeted particle size of ≤70 nm and PDI value of <0.3 by superimposing the responses obtained during the optimization as shown in **Fig. S3**. In the contour plots **(Fig S3)**, the design space is represented in yellow color portions, while the grey segment represents the outlier background region. This means that any combination of experimental process parameters in yellow design space region theoretically produces the desired experimental outcome. While any point in the grey outlier background region does not produce the product fitted to criteria of the quality product[6].

Suggestively, the design space with a higher probability of attaining product with desired product attribute (Desirability ≈1) has been obtained using the solution suggested in contour plots (Albumin concentration: 4% w/v; Ethanol concentration: 100 %v/v; Agitation speed: 1000 rpm) that is predicted to yield nanoplex of particle size 67.044 nm and 0.268 PDI **(Table S3** and **Fig. S3)**.

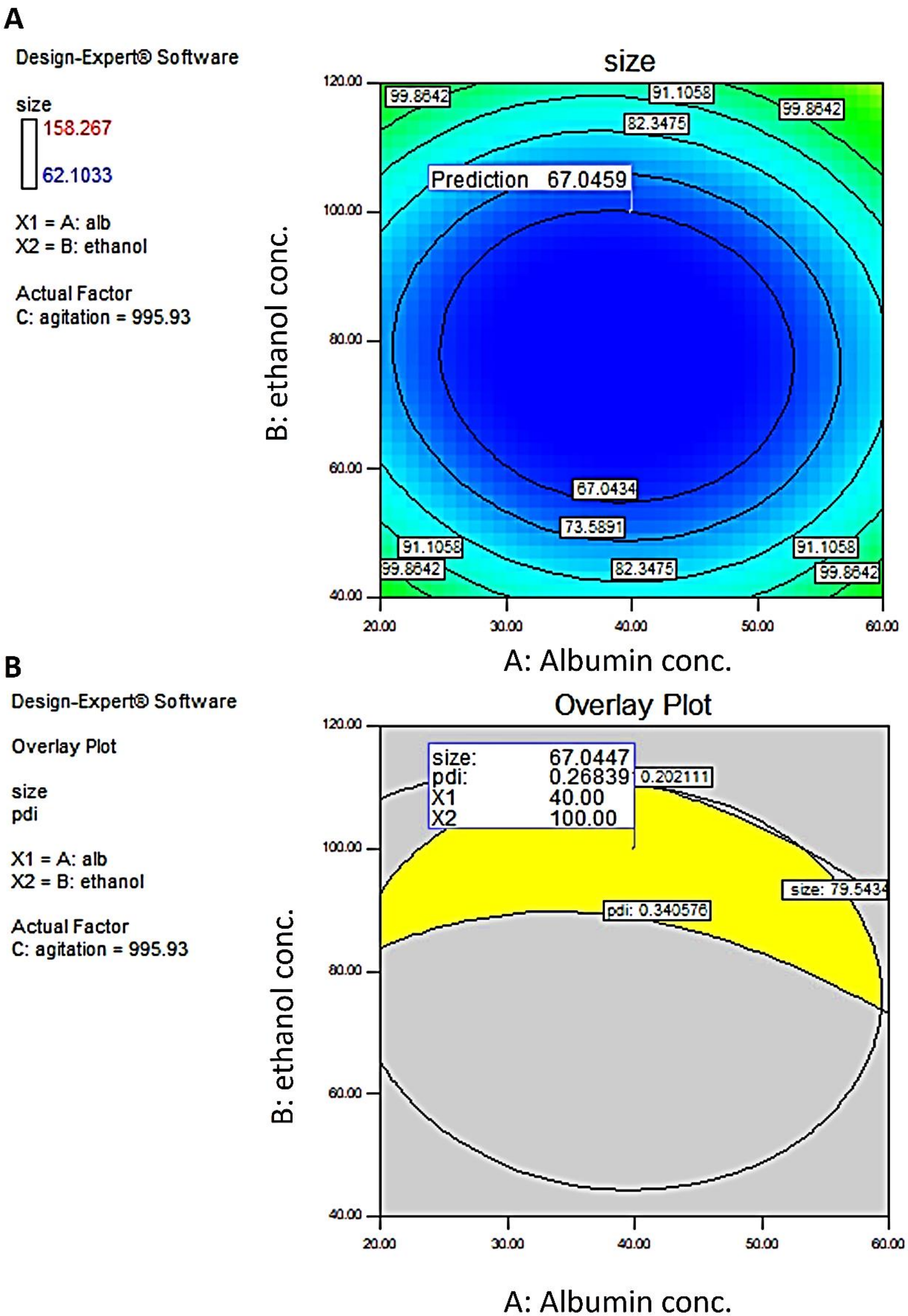

Supplementary Figure S3. (A) Contour plot with design space, and (B) Overlay plot for the synthesis of nanoplex with target product attributes

Supplementary Table S3: The validation batch with predicted and observed response of nanoplex according to desirability factor.

Response	Value predicted by QbD software	Practical value	Standard deviation	% Error
Particle size (nm)	67.044	66.23	5.29	0.27
PDI	0.268	0.289	0.031	7.83
**Reaction condition:** *Albumin concentration: 4% w/v; Ethanol concentration: 100 %v/v; Agitation speed: 1000 rpm.* Results are represented as means of at least ten individual experiments *(n=10).*				

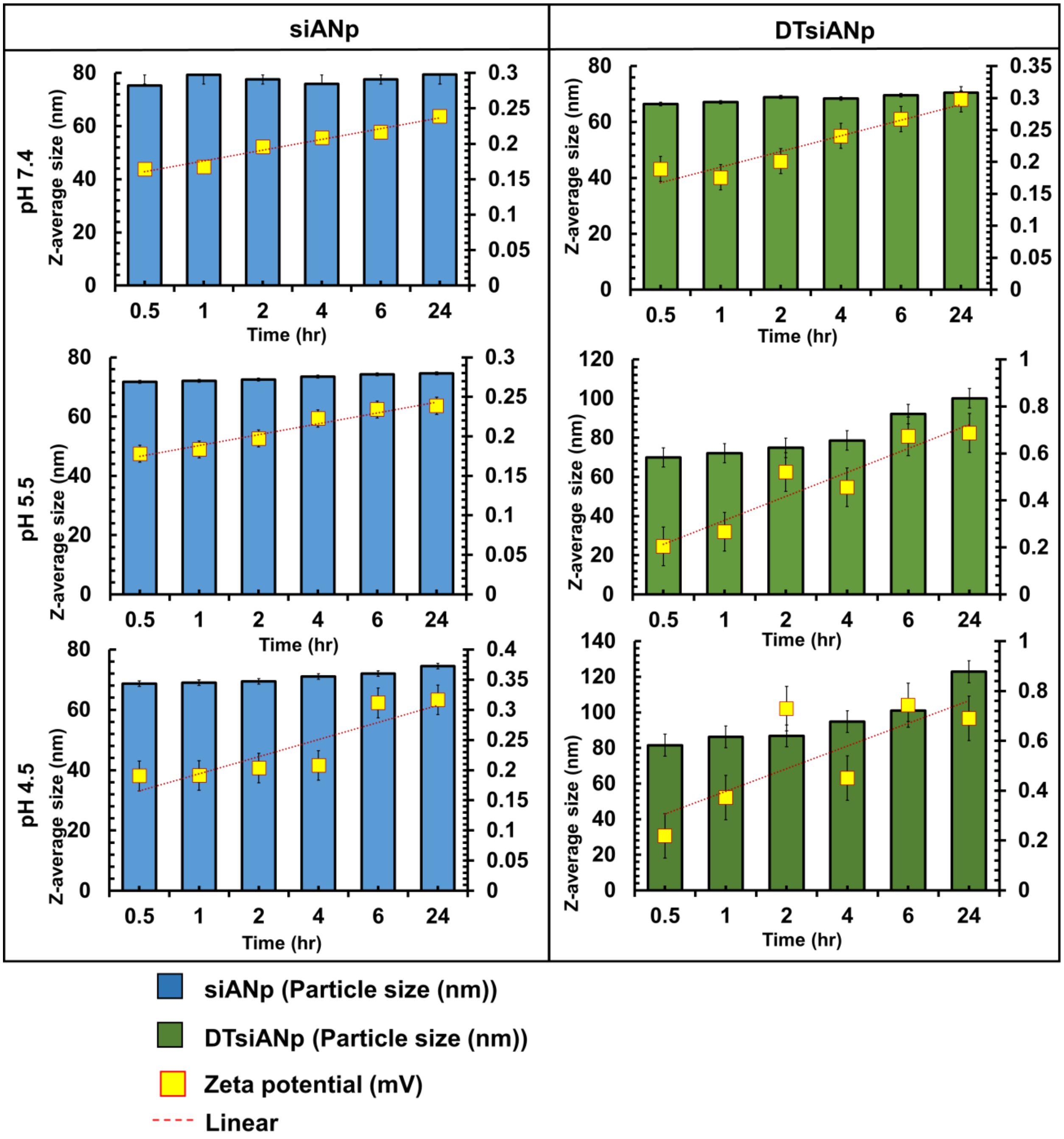

Supplementary Figure S4. The effect of pH 7.4, pH 5.5 and pH 4.5 on particle size at various time points. The nanoplex are found to be stable at various pH up to 24 hr. Results are represented as mean±S.D *(n=3).*

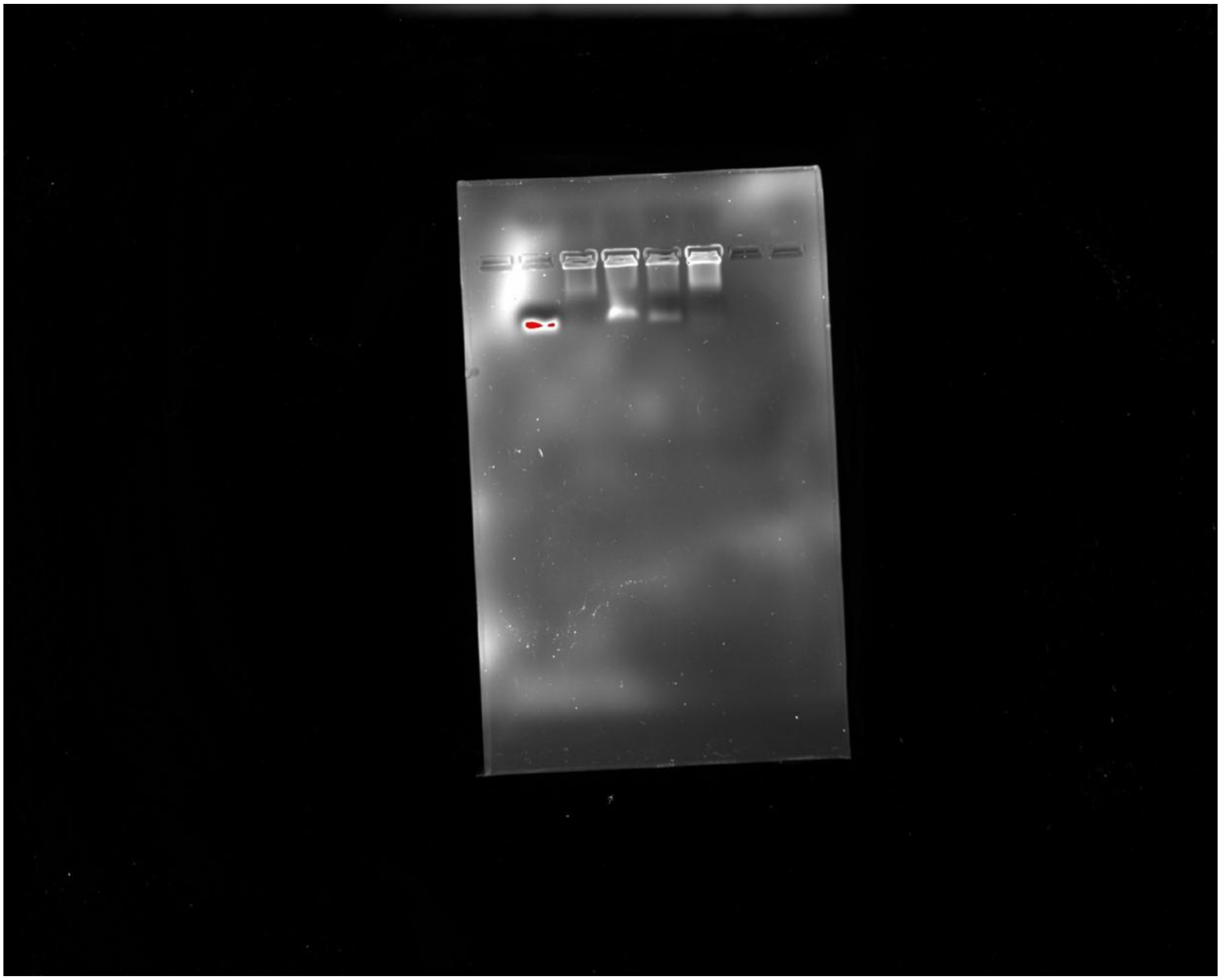

Supplementary Figure S5. Original Gel for Figure 2E of the main manuscript. Well-description (Left to right) Lane 1: Vacant; Lane 2: Lane 3: siANp after centrifugation; Lane 4: siANP after centrifugation (supernatant); Lane 5: DTsiANp after centrifugation (supernatant); Lane: 6 DTsiANp after centrifugation; Lane 7: Vacant; Lane 8: Vacant

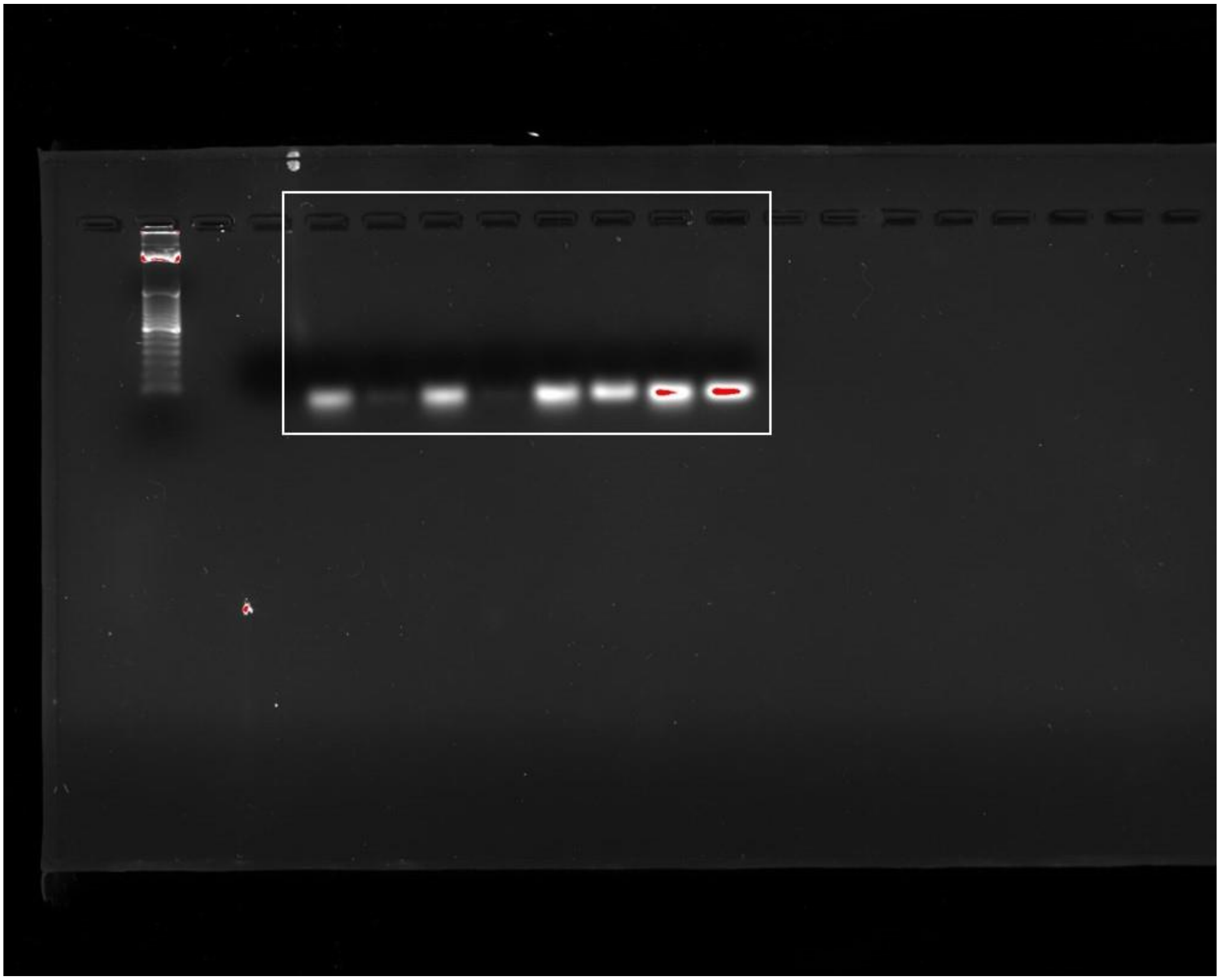

Supplementary Figure S6. Representing Gel electrophoresis for the selection of *d*:siR complexation condition. The cropped portion of gel as shown in the inset has been presented in the main manuscript as Figure 1B. Well description (Left to right) Lane 1: positive control having siRNA in equivalent amount as in *d*:siR complex prepared at 1 *n/p* ratio; Lane 2: *d*:siR complex prepared at 1 *n/p* ratio; Lane 3: positive control having siRNA in equivalent amount as in *d*:siR complex prepared at 0.5 *n/p* ratio; Lane:4: *d*:siR complex prepared at 0.5 *n/p* ratio; Lane 5: positive control having siRNA in equivalent amount as in *d*:siR complex prepared at 0.25 *n/p* ratio; Lane 6: *d*:siR complex prepared at 0.25 *n/p* ratio; Lane 7: positive control having siRNA in equivalent amount as in *d*:siR complex prepared at 0.12 *n/p* ratio; Lane 8: *d*:siR complex prepared at 0.12 *n/p* ratio.

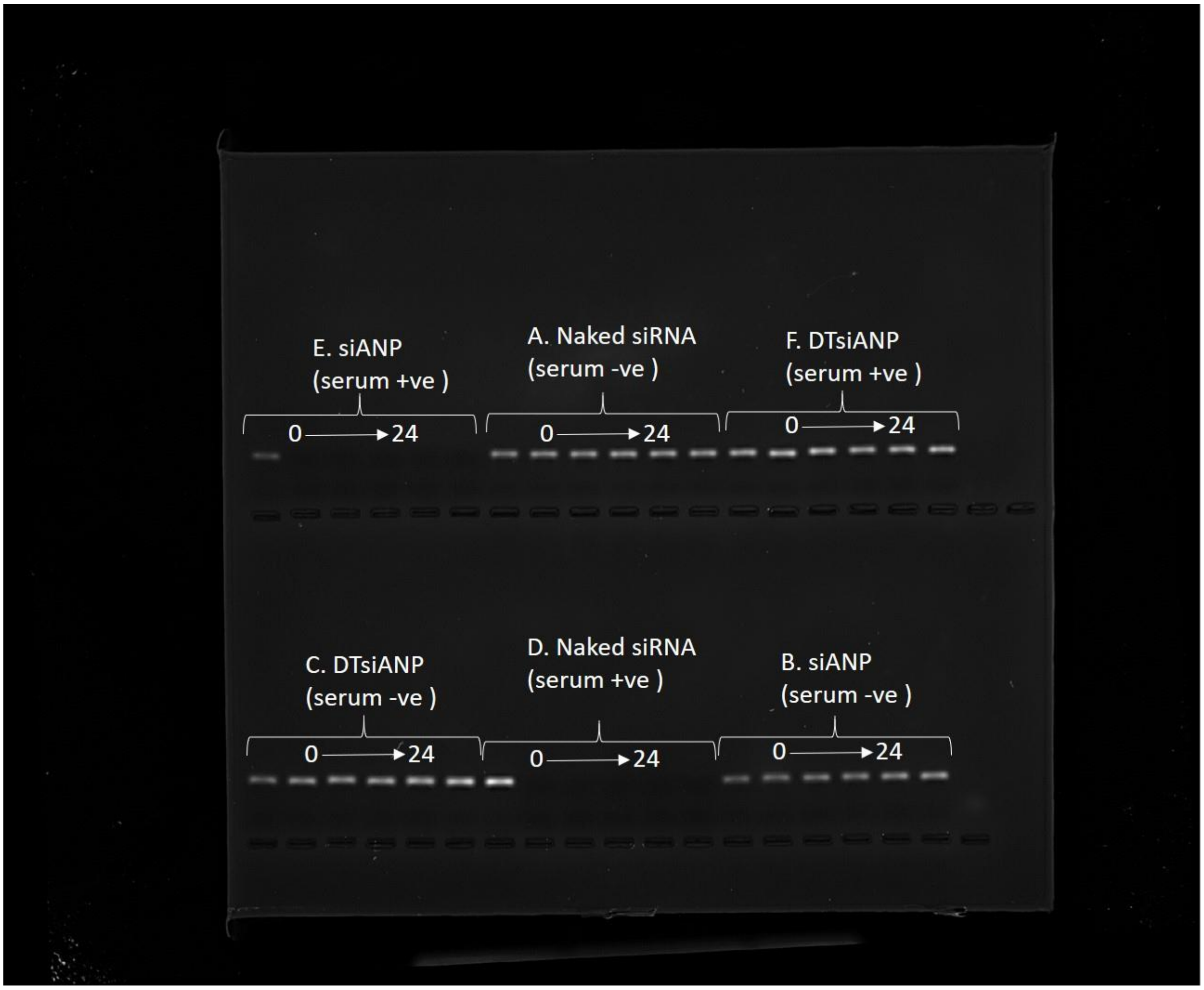

Supplementary Figure S7. Stability profile of siRNA in absence of serum [Serum (-ve)] and in presence of serum [Serum (+ve)] as evaluated by gel electrophoresis (A) naked siRNA [Serum (-ve)], (B) siANp [Serum (-ve)], (C) DTsiANp [Serum (-ve)], (D) naked siRNA [Serum (+ve)] (E) siANp [Serum (+ve)], and (F) DTsiANp [Serum (+ve)]. The inset of this gel has been presented in Figure 4A-F of the main manuscript.

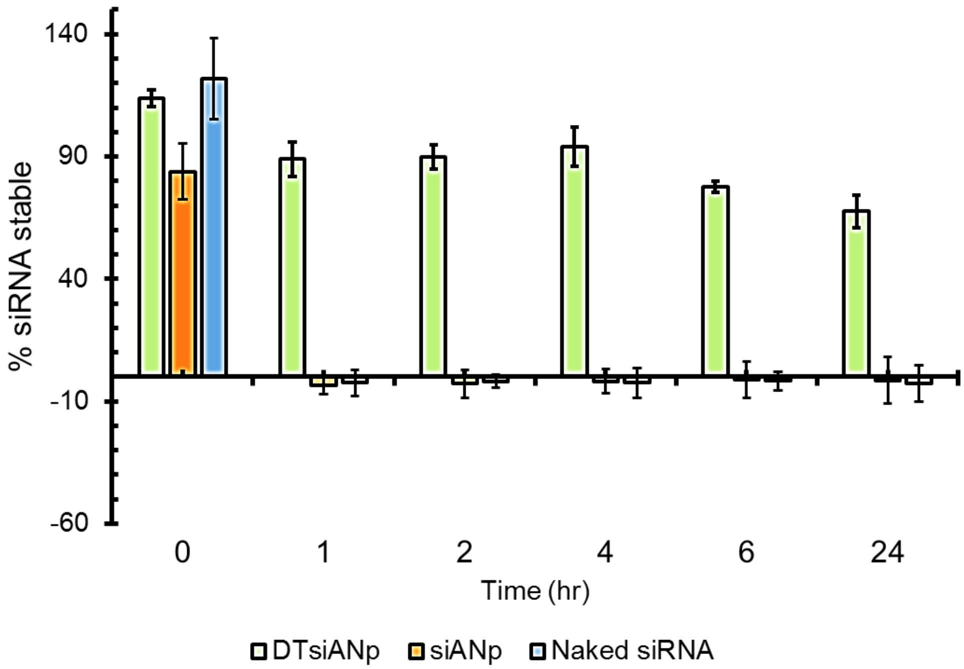

Supplementary Figure S8. Percentage siRNA stability after treatment with the serum comprised media. The percentage stability was calculated from the serum treated group's siRNA band intensity with reference to siRNA band intensity of serum untreated group.

Assessment of albumin purity. The albumin was procured from the HiMedia Laboratories GmbH (Germany; Distributor: Mumbai, India). As suppliers report, the fraction V albumin was ≥96 % pure and does not contain any other component from the fraction V. The report also confirms that the used albumin was free from IgG, fatty acids and proteases. The same grade of albumin has been widely utilized as a standard for several ELISA assays as reported in datasheet of the manufacturer (http://himedialabs.com/TD/TC194.pdf)[7].

Further, we have also assessed the purity of albumin through SDS-PAGE for the evaluation of components from the fraction V albumin. Results of SDS PAGE *(n=6)* confirms the absence of any impurity and the albumin sample was showing the albumin associated bands between 50–75 KDa. The representative image of the gel has been presented in the supplementary file and can be seen as **Supplementary Figure S9**. Our result from SDS-PAGE was in good agreement with the reported literature[8-10]. The purity of albumin was also analyzed by determining its net protein content through BCA assay kit method. The result obtained using the 1 mg/ml albumin solution showed the net albumin concentration of s 0.969 mg/ml, which confirmed the purity reported by

the manufacturer (supplementary file as **Figure S10).** These the experimental outcomes infer that the albumin used in this investigation was pure and was not containing any component from the fraction V albumin.

The proteomic analysis of Fraction V albumin was also performed using MALDI-TOF/MS (AB Sciex 5800 TOF/TOF MALDI; matrix: Sinapinic Acid (30% ACN); albumin (1 mg/100 uL (30% ACN); sample: matrix= 1:5). A peak was found with m/z 66,265.1484 Da (+1) corresponds to albumin and another peak at 33177.0430 Da (+2) and these two characteristic peaks for the albumin. The result also confirmed the albumin and purity of albumin as well as affirmed the absence of any other component of fraction V albumin. The result can be seen in the supplementary data as **Figure S11**. The data obtained in our investigation was in good agreement with the published literature[11]

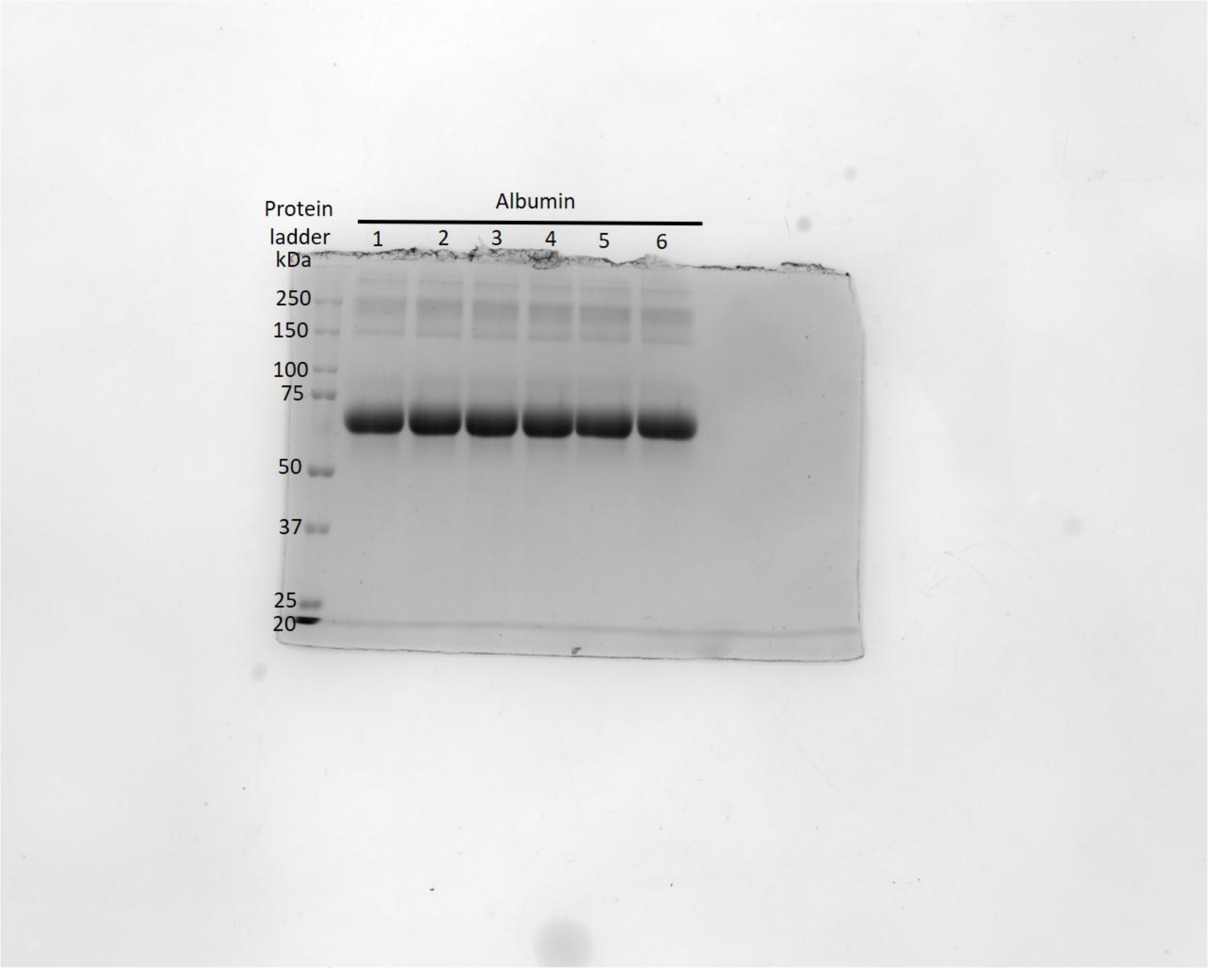

Supplementary Figure S9. SDS-PAGE performed on albumin (15 µM). Protein ladder showing band for different molecular weight (kDa). Lane 1–6: albumin (n=6).

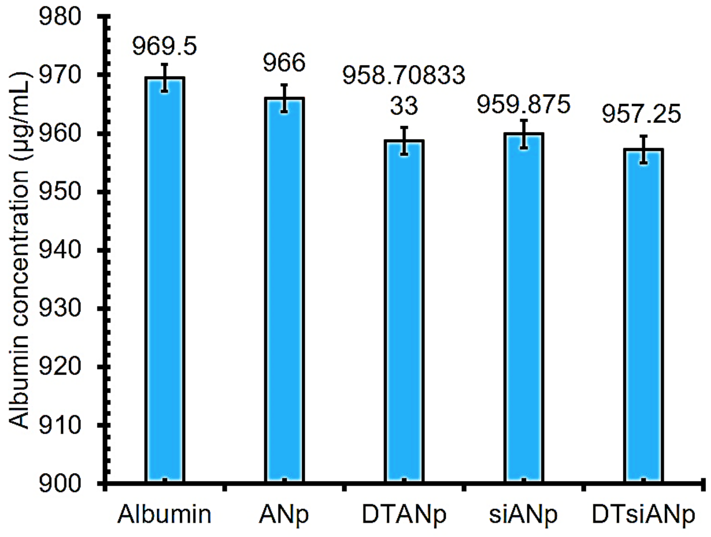

Supplementary Figure S10. Protein concentration determination of albumin via BCA assay kit for albumin, albumin nanoplex (ANp), siRNA loaded albumin nanoplex (siANp), dendrimer templated albumin nanoplex (DTANp), dendrimer templated and siRNA loaded albumin nanoplex (DTsiANp). Results are represented as mean±S.D (n=3).

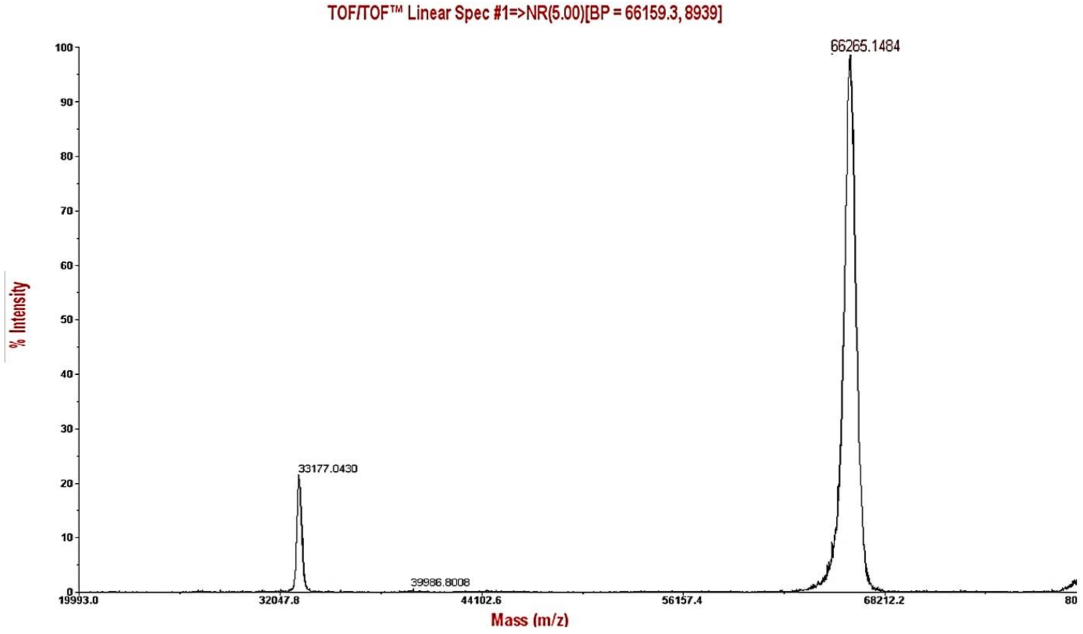

Component	Charge (n)	(M+nH) n+
Albumin fraction V	+1	66265.1484
	+2	33177.0430

Supplementary Figure S11. Representative spectrum for Albumin obtained using MALDI-TOF MS/MS. Table suggesting molecular mass peak of albumin. (AB Sciex 5800 TOF/TOF MALDI; matrix: Sinapinic Acid (30% ACN); albumin (1 mg/100 uL (30% ACN); sample:matrix= 1:5).

Confirmation of albumin presence in nanoplex. The presence of albumin in ANp, DTANp, siANp, and DTsiANp was confirmed via SDS-PAGE with reference to plain albumin. Result confirmed that the albumin remained intact even after formulating albumin as nanoplexes (ANp, DTANp, siANp, and DTsiANp). The obtained bands of albumin from ANp, DTANp, siANp, and DTsiANp have remained at similar molecular weight with reference to plain albumin (75-55 kDa; **Supplementary Figure S12)** confirmed that albumin remained in the intact form even after the preparation of nanoplex.

Further, the presence of albumin in ANp, DTANp, siANp, and DTsiANp was also confirmed via protein concentration determination via BCA reagent assay kit. The protein concentration obtained from 1 mg/mL nanoplex sample was found to be 0.966±0.032 mg/ml (ANp), 0.958±0.043 mg/ml (DTANp), 0.959±0.012 mg/ml (siANp), and 0.957±0.039 mg/ml (DTsiANp), respectively (**Supplementary Figure S10**). The data confirmed that the albumin composed of a major portion of nanoplexes as 96.6±0.92 % (ANp), 95.8±3.27 % (DTANp), 95.96±1.23 % (siANp) and 95.76±4.24 % (DTsiANp), respectively.

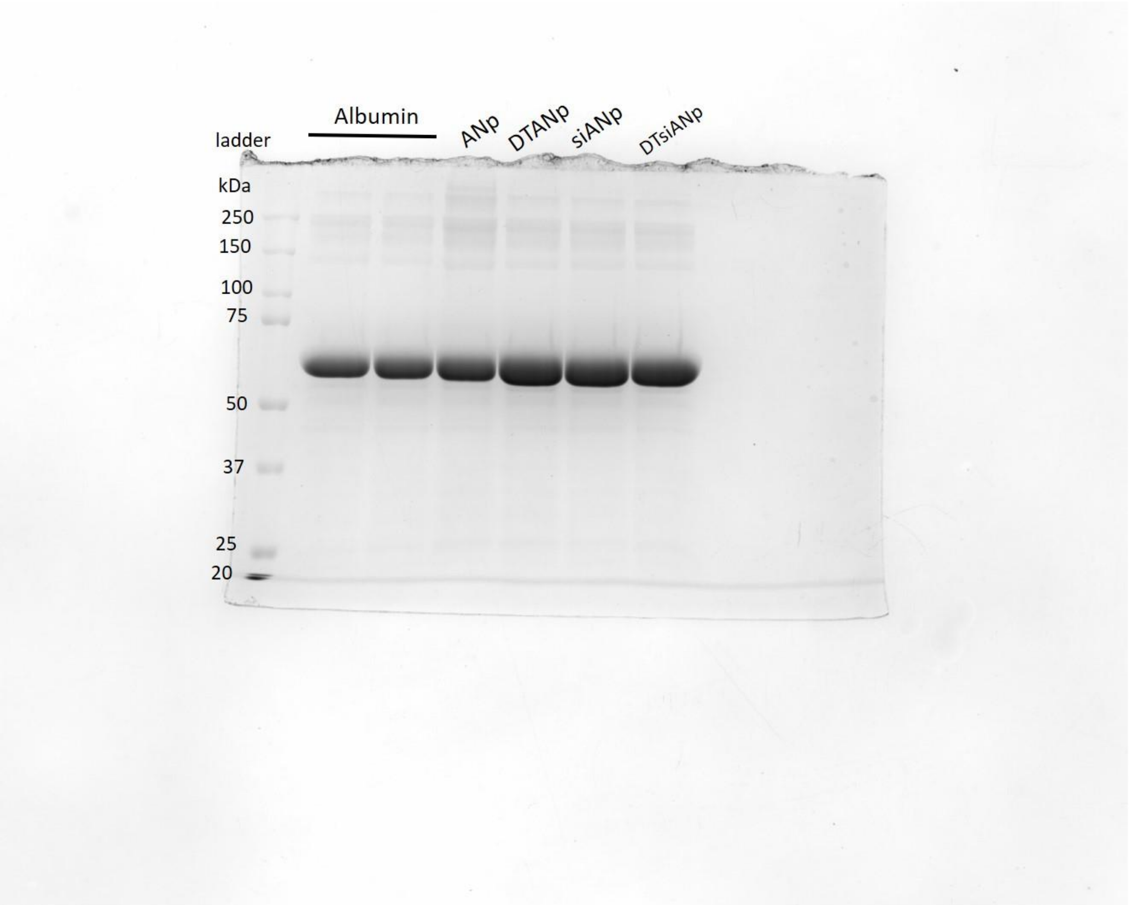

Supplementary Figure S12. SDS-PAGE performed on albumin (15 µM) and albumin formulations viz. ANp, DTANp, siANp, and DTsiANp. Protein ladder showing band for different molecular weight (kDa). Lane 1-2: albumin, lane 3: ANp, Lane 4: DTANp, lane 5: siANp, lane 6: DTsiANp.

Confirmation of dendrimeric template presence in nanoplex. The incorporation of dendrimer molecule in the nanoplex was confirmed via 2,4,6-Trinitrobenzene Sulfonic Acid (TNBSA) assay. This assay gives a quantitative analysis of the free primary amino group in a given sample. The outcome of this experiment suggested the presence of $88.905 \times 10^{23} \pm 9.01$ free primary amino group per gram of plain albumin sample. The nanoplex showed $90.66 \times 10^{23} \pm 4.24$ free primary amino group per gram of ANp, $93.87 \times 10^{23} \pm 3.70$ per gram of siANp. Whereas, the obtained no. of number of free primary amino group from DTANp and DTsiANp was found to be $117.92 \times 10^{23} \pm 5.09$ and $121.2 \times 10^{23} \pm 2.51$ per gram of nanoplex, respectively (**Supplementary Figure S13**). An enhancement of 23.11±1.08 % and 22.54±1.67 % in the proportion of the free primary amino group was observed in case of DTANp and DTsiANp. The increment in the proportion of primary amino group in the dendrimeric templated nanoplex can be ascribed to the presence of dendrimer in the DTANp and DTsiANp formulation.

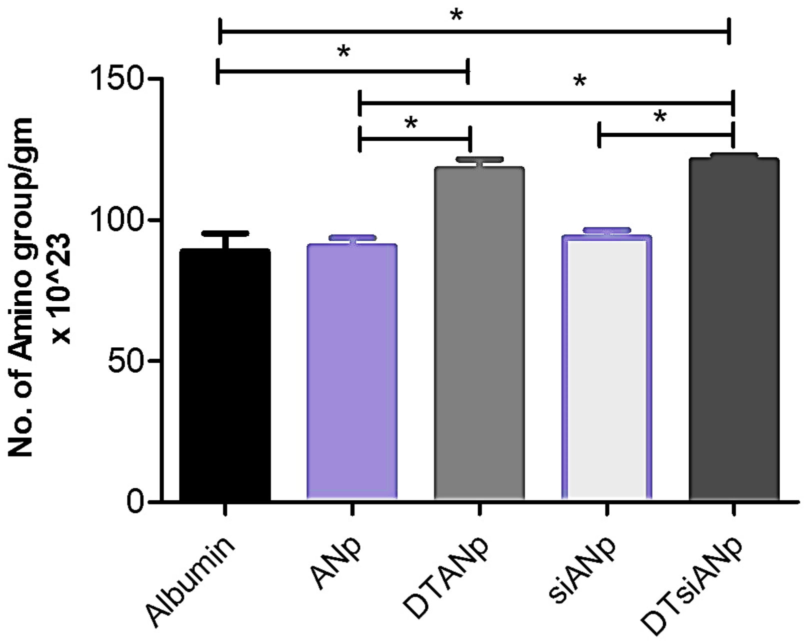

Supplementary Figure S13: 2,4,6-Trinitrobenzene Sulfonic Acid (TNBSA) assay for albumin, ANp, DTANp, siANp, and DTsiANp. *p<0.05. Results are represented as mean±S.D (n=3).

Further, the presence of dendrimeric template in the nanoplex was also confirmed through surface charge analysis. The surface charge of the plain albumin, ANp and siANp was found to be –25.6±0.95 mV, –27.5±0.64 mV, and –26.8±0.89 mV, respectively. After the incorporation of the dendrimeric template in the nanoplex, the surface zeta potential changed to –16.3±1.27 mV and –16.1±1.06 mV for DTANp and DTsiANp, respectively. This 40.72±1.42 % and 39.92±1.16 %, respective increment in the surface charge of DTANp and DTsiANp compared to ANp and siANp suggested the incorporation of dendrimeric template in nanoplex. The **Supplementary Figure S14** data evidenced that the presence of albumin and dendrimer molecule in formulated nanoplex.

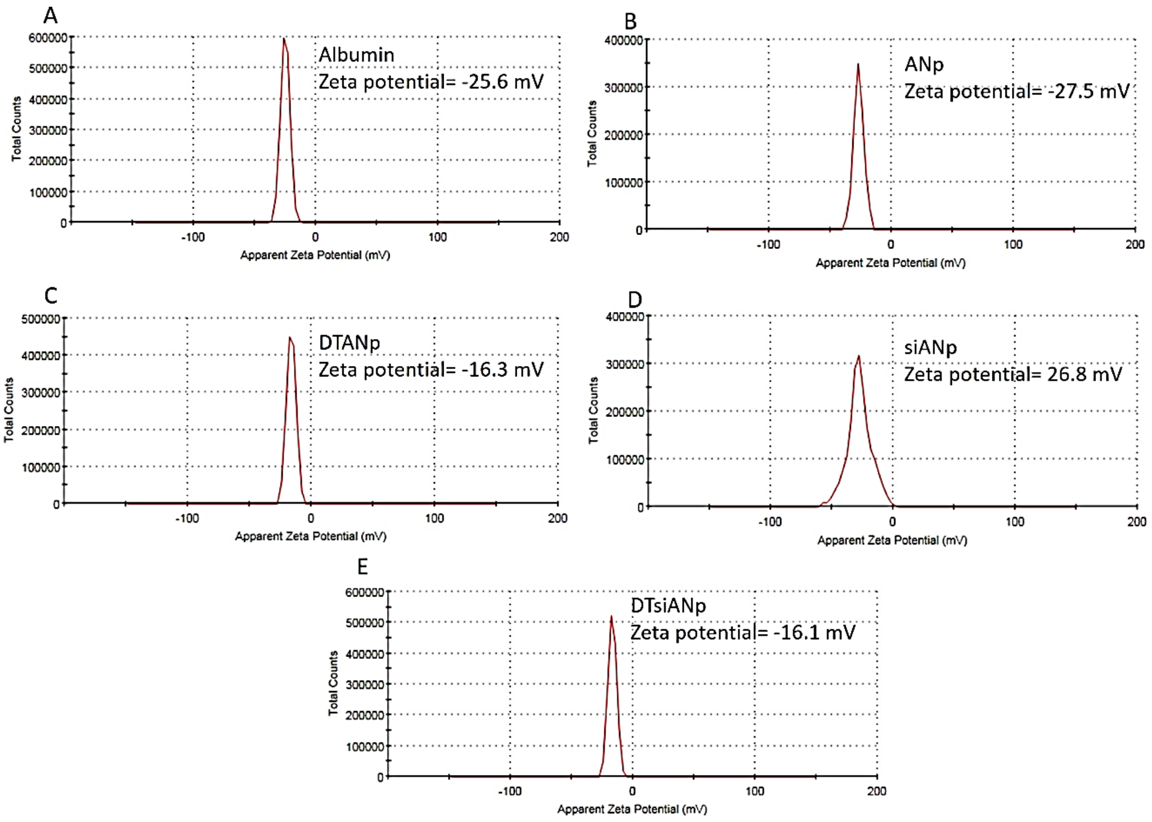

Supplementary Figure S14: Surface zeta potential of (A) albumin (B) ANp (C) DTANp (D) siANp (E) DTsiANp.

RNase protection assay. The results of this study suggested that the precipitated/loosely bound/surface adhered siRNA from siRNAp and DTsiANp gets degraded in presence of RNase enzyme which can be seen in lane:2 and lane:3, respectively. Here, lane:1 act as a control free siRNA of equal amount (150 ng siRNA/well). Whereas, the encapsulated siRNA gets released out after sonication and repeated vigorous pipetting was found to be intact that can be seen as lane:4

and lane:5, respectively **(Supplementary Figure S15)**. It advocates that when intact pellet was treated with RNase enzyme, then only the loosely bound siRNA or surface adhered siRNA gets degraded. In this case, the band of siRNA does not appear. The emergence of the band after forceful liberation of siRNA (lane:4 and lane:5) infers that the major portion of siRNA is available in the encapsulated form inside the nanoplex in a stable form. In case of the lane: 4, a faint band of siRNA was observed due to very less (~15% siRNA) entrapment of siRNA in siANp owing to the negative charge of albumin. Besides, lane: 6 and lane: 7 were not showing any siRNA band because in these cases the siRNA was simply precipitated with albumin and albumin-dendrimer solution, respectively. It may be noted that when the precipitated siRNA directly comes in contact with RNase enzyme, siRNA gets degraded easily (lane:6 and lane:7). Whereas encapsulated siRNA remains intact inside nanoplex carrier and protect siRNA from degradation (lane:4 and lane:5; also proved through serum stability study). This experiment confirms that the siRNA was actually loaded inside the nanoplex and are formed nanoplex was not having the physical co-precipitates of siRNA and albumin.

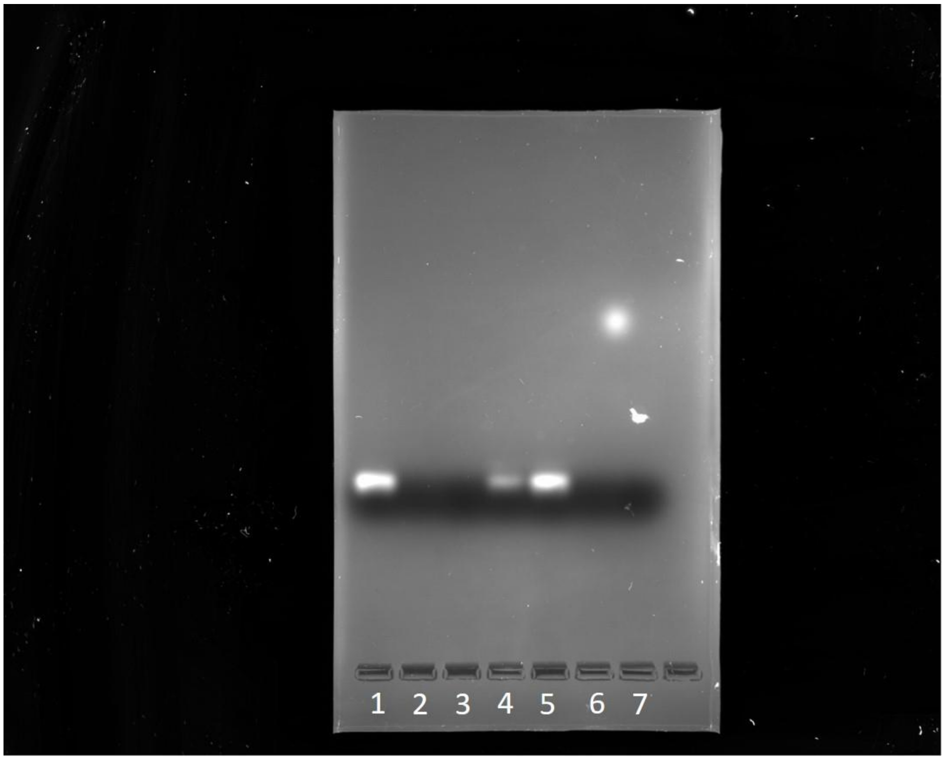

Supplementary Figure S15: Agarose gel electrophoresis data representing actual incorporation of siRNA in nanoplex. Lane 1: free siRNA, Lane 2: siANp (pellet+sonication)+RNase, lane 3: DTsiANp (pellet+sonication)+RNase, lane 4: siANp+RNase, lane 5: DTsiANp+RNase, lane 6:

Albumin+siRNA external precipitation (pellet)+RNase, lane 7: Albumin+dendrimer+siRNA external precipitation (pellet)+RNase**.**

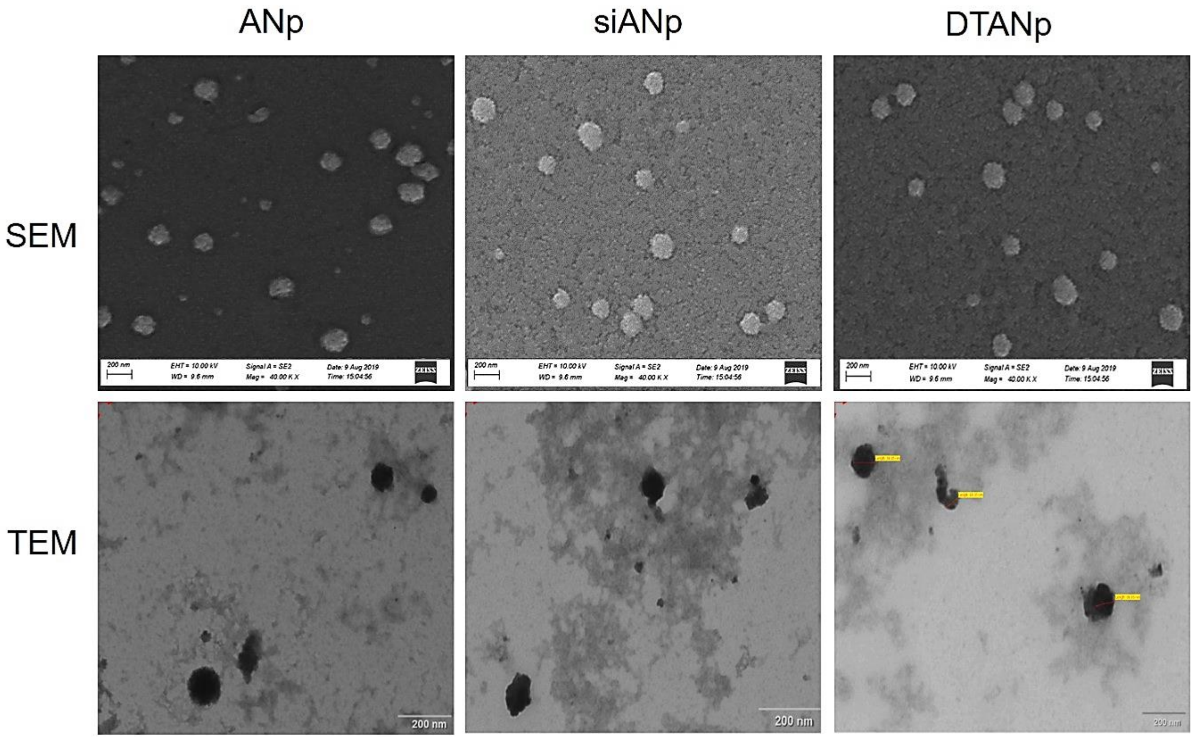

Supplementary Figure S16: Representative SEM images (A) ANp (B) DTANp (C) siANp, and representative TEM images (D) ANp (E) DTANp (F) siANp.

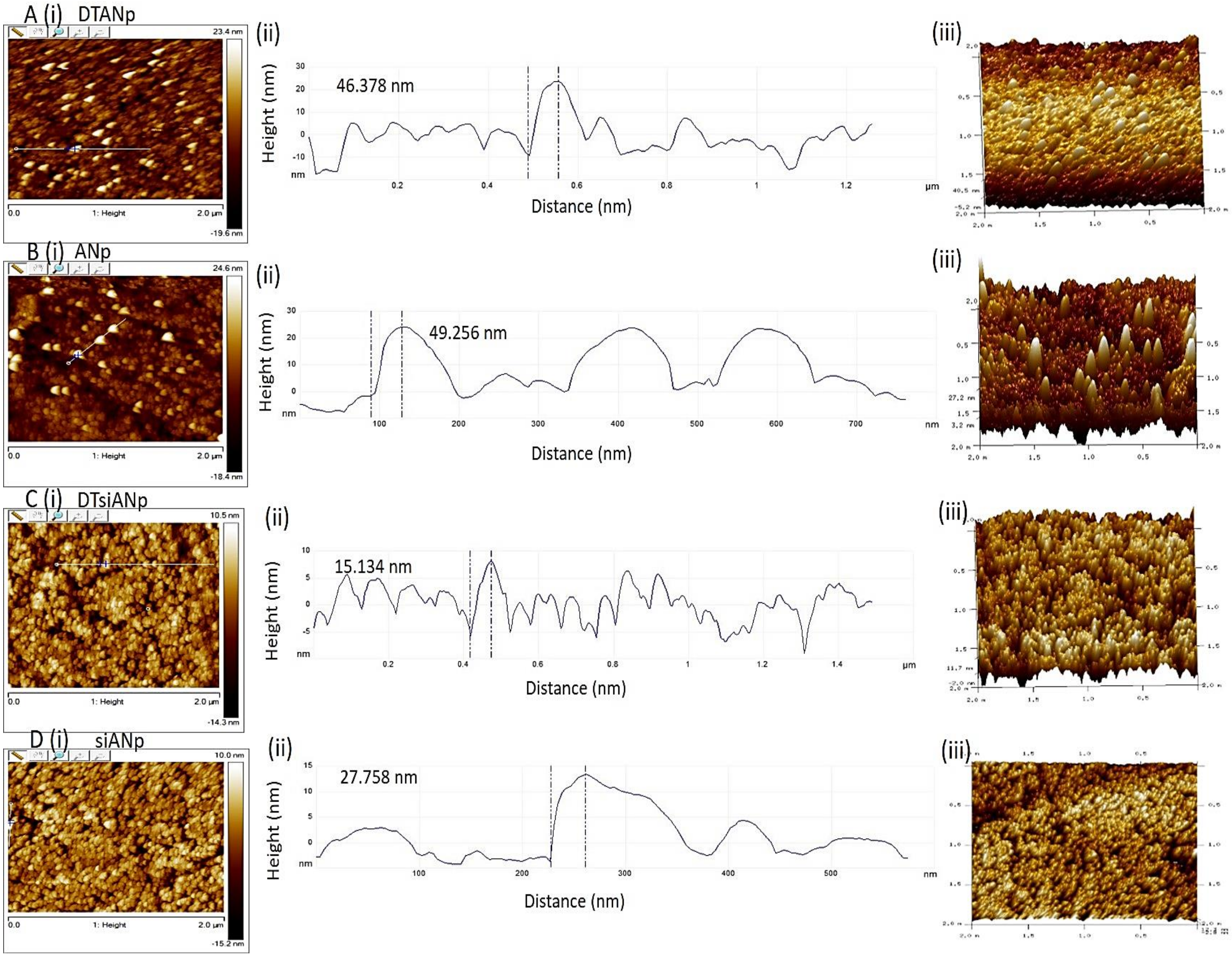

Supplementary Figure S17: Representative images of AFM analysis for (A) DTANp, (B) ANp, (C) DTsiANp and (D) siANp; (i) 2D height images of nanoplex, (ii) respective sectional profile (iii) 3D height images of nanoplex. Scale bar represents 2 µm.

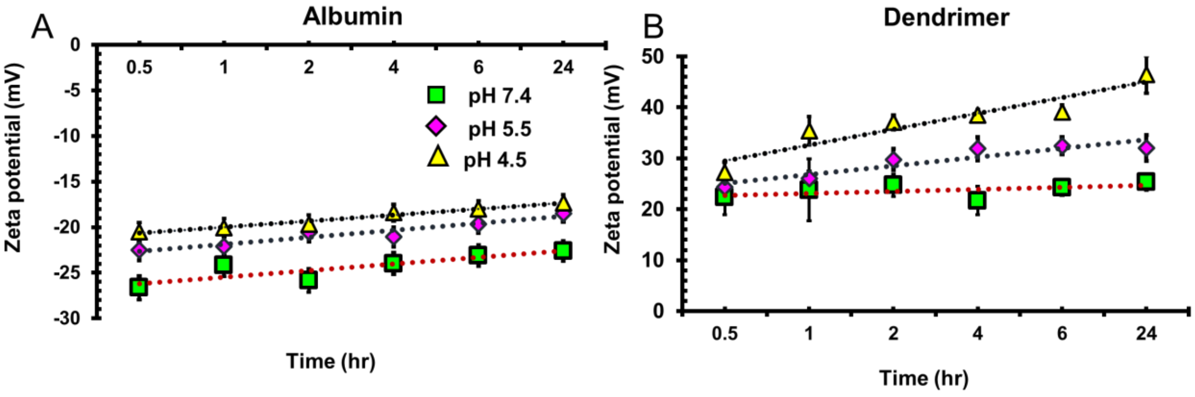

Supplementary Figure S18: pH responsiveness of (A) plain albumin and (B) dendrimer in terms of surface charge at pH 7.4, 5.5 and 4.5.

Endo/lysosomal escape assay. As observed in **Supplementary Figure S19**, after 12 hr, the siANp was not exhibiting any cytosol associated fluorescence. The fluorescence from siANp was primarily restricted to endosome inferring the lack of endosomal escape tendency in this nanoplex. Whereas, DTsiANp showed a significant co-localization of green fluorescence of FAM-siRNA and red fluorescence of endosome selective Lyso-Tracker dye at 8 hr. Initially at 6 hr, very less red fluorescence was observed compared to at 8 hr, which signifies the endosomal uptake of DTsiANp. Notably, at 12 hr time point, the red fluorescence shifted to the cytoplasmic compartment of the cell, and only the green fluorescence was observed in the endosome. This event clearly signifies the endosomal escape ability of DTsiANp (**Supplementary Figure S19).**

The endosomal escape tendency of DTsiANp can be ascribed the pH-responsive behavior of DTsiANp owing to the existence of free primary amines in dendrimeric template present in the nanoplex. It may be noted that the amine groups of dendrimers undergo protonation under acidic pH leading to enhancement in net surface positive charge. The protonation of the nanoplex assembly under the acidic environment of endosome generates the repulsive microenvironment in the architect of nanoplex. This repulsive microenvironment existing within the nanoplex leads to an increment in its hydrodynamic crevices volume with a marked increase in ionic concentration osmotically. The cumulative enhancement in size of DTsiANp leads to the swelling of the endosomal compartment ultimately leading to the rupture of the endosomal membrane to mediate endosomal escape of DTsiANp. This event liberates the DTsiANp from the endosomal compartment before its degradation by lysozyme (phenomenon referred as endosomal escape).

This effect was further confirmed via pH-responsive change in the surface zeta potential and particle size of nanoplex **(Supplementary Figure S19)**. This pH-responsive and endosomal escape effect was primarily due to dendrimeric template of nanoplex not because of the loaded siRNA. It confirmed that pH has no notable impact on siRNA. The observed effect can be ascribed to the protonation behavior of dendrimeric template present in the nanoplex[12].

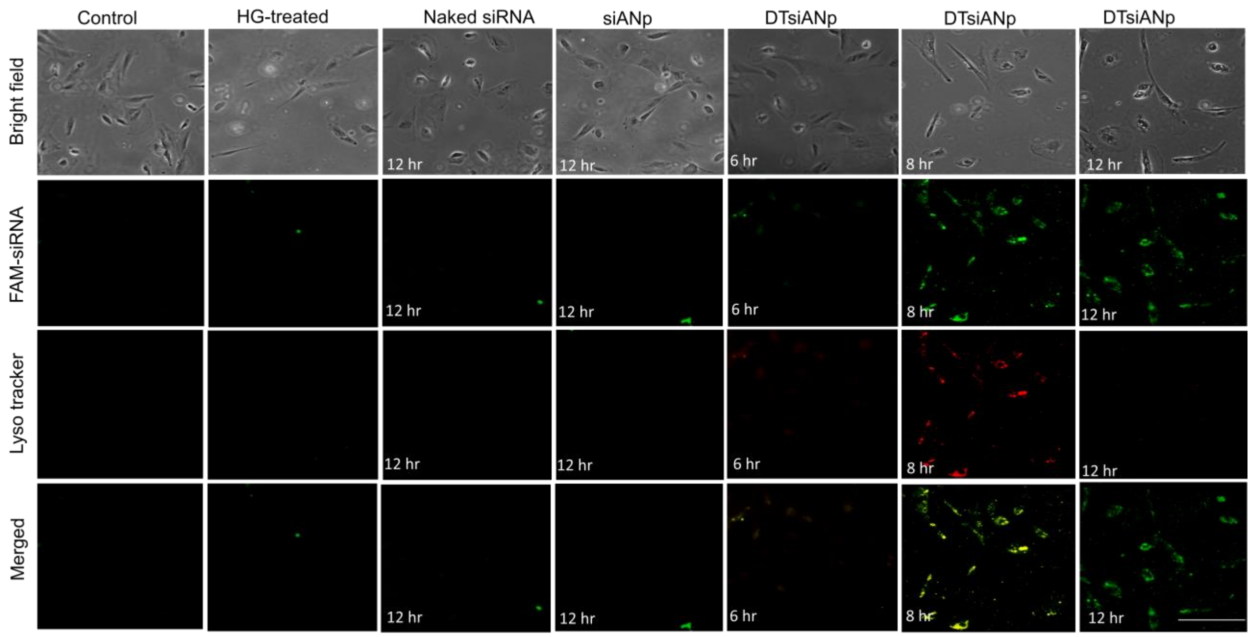

Supplementary Figure S19: Images representing endosomal escape activity. Green fluorescence was observed from FAM siRNA loaded in DTsiANp and red fluorescence was observed from endo/lysosome labeled with lyso-tracker. Co-localization of FMA-siRNA with lyso-tracker showed yellow fluorescence. Scale: 25 µm.

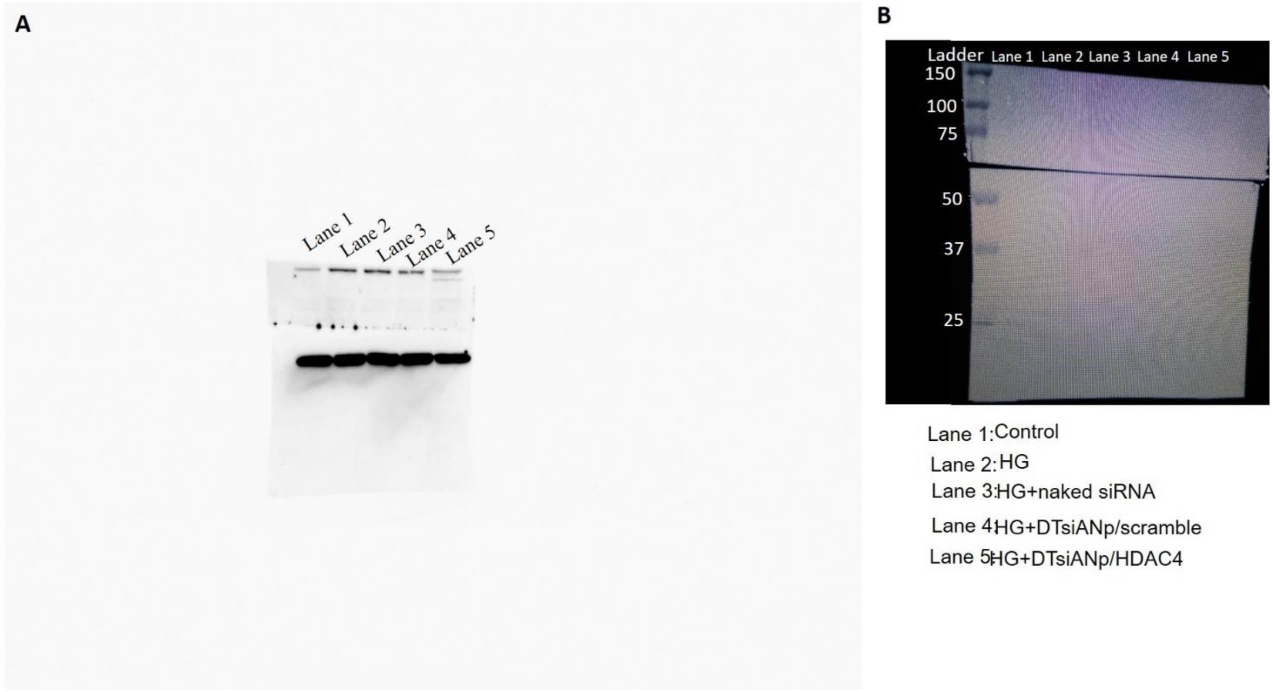

Supplementary Figure S20. (A) Western blot analysis for HDAC4 protein and β-actin expression in HG-treated podocytes from the same blot and from the same membrane. HG-treated cells were taken as a positive control. (B) Representative blot image for the ladder indicating the molecular weight of protein and lane explanation; Lane1: Control; lane 2: HG; Lane 3: HG+naked siRNA;

Lane 4: HG+DTsiANp/Scramble; Lane 5: HG+DTsiANp/HDAC4. The inset of this blot has been presented in Figure 5D of the main manuscript.

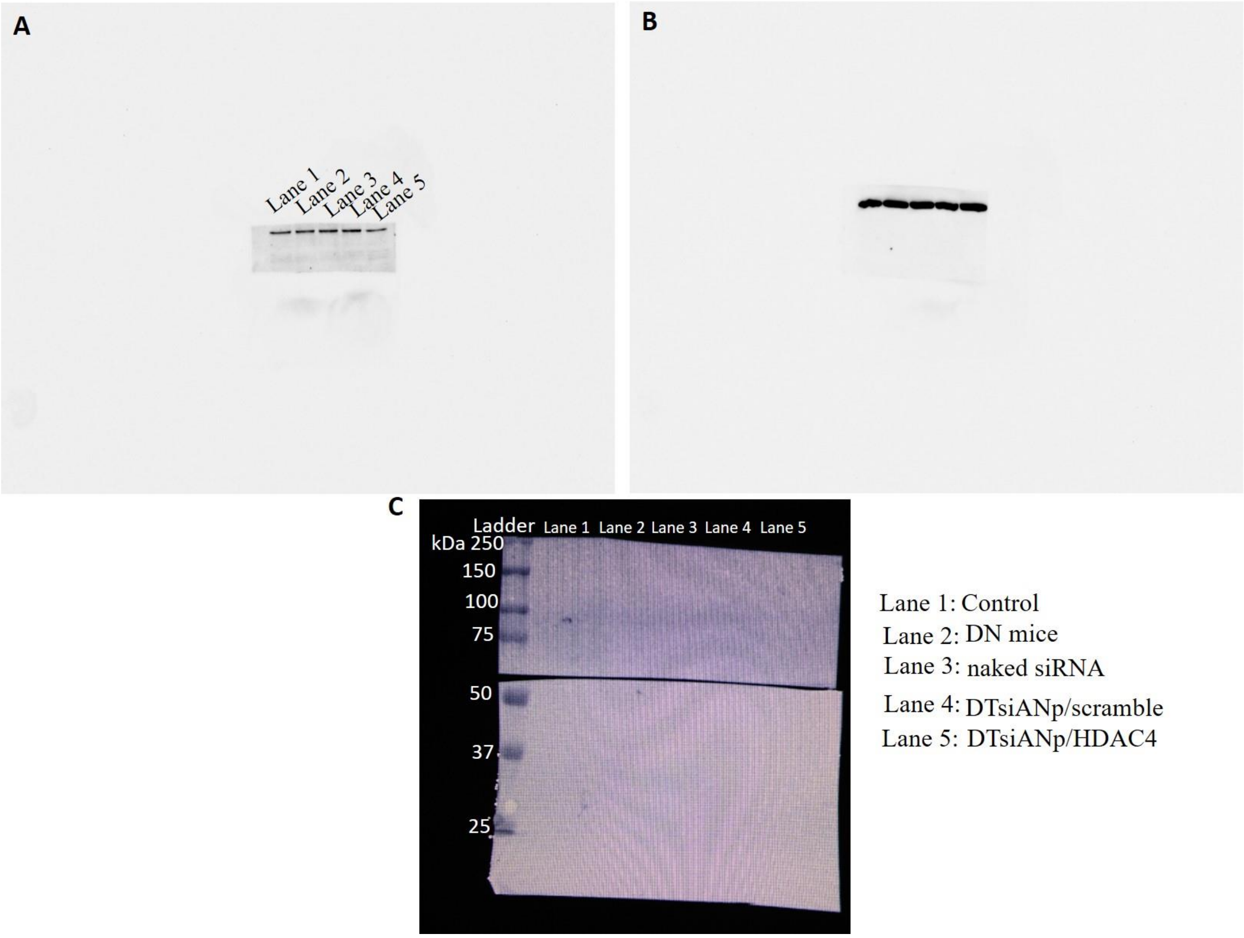

Supplementary Figure S21. (A) and (B) Western blot analysis for HDAC4 protein and β-actin expression in healthy control and DN diseased kidneys obtained from the same blot and from the same membrane (C) Representative blot image, ladder indicating molecular weight of protein and lane explanation; Lane1: Control; lane 2: DN mice; Lane 3: naked siRNA; Lane 4: DTsiANp/Scramble; Lane 5: DTsiANp/HDAC4. The inset of this blot has been presented in Figure 6D of the main manuscript

Methods

Preliminary screening of process variables using Two-level Five-factor (2^5) full factorial design. The product as well as process-related parameters that directly effects the net properties of nanoplex and its probable effect on critical quality attributes were the first identified[13]. Based on scientific understanding, for the therapeutic efficacy and targeting purpose given parameters are substantially contribute towards targeting as well as therapeutic efficacy. For the purpose of optimization, we applied Two-level factorial design for identification of main affecting factors from a large number which critically affects the formulation quality. Factors were estimated at two levels (five factors) as high level (+1) and low level (-1) and level of parameters were decided based on preliminary experiments and scientific literature data and. This high- and low-level independent variables were as shown in **Table S4** Design-Expert Software (Stat-Ease, Version 7.0; Minneapolis, MN) software was utilized which produce randomize design matrix of experiments which were performed in a random manner. Analysis of applied design by means of regression analysis, ANOVA and coefficients of quadratic equations were taken into consideration for particle size and PDI.

Supplementary Table S4: Factors and corresponding process parameter levels for two-level full factorial design

Factor	Unit	Process Parameter Level	
		Low	High
Albumin concentration	%w/v	2	6
Ethanol volume	%v/v	200	600
Agitation speed	rpm	500	1000
Ethanol concentration	%v/v	40	100
Rate of addition	µl/min	20	60

The mentioned factors which affect nanoplex preparation and were taken in their low and high levels were based on previously run experiments.

QbD-driven Optimization of screened process parameters using Box-Behnken design. After the process and product parameters using two-level full factorial design, Box-Behnken design was applied using Design-Expert Software (Stat-Ease, Version 7.0; Minneapolis, MN) for final optimization of selected process parameters. The response surface method was applied using three selected factors including albumin concentration, ethanol concentration, and agitation speed (Total

17 runs). It may be noted that the low and higher level (five-factor) were directly taken from two-level full factorial design and mid-level were at middle-point of low and high levels as shown in **Table S5**.

The Box-Behnken design gave polynomial equations for determining the predictive interactions among the selected process parameters. With the help of design, a non-linear quadratic model was generated and the polynomial equation was obtained as follows:

$Y = \beta_0 + \beta_1 A + \beta_2 B + \beta_3 C + \beta_{12} AB + \beta_{13} AC + \beta_{23} BC + \beta_{11} A^2 + \beta_{22} B^2 + \beta_{33} C^2$……………………………………………………..Equation (S3)

Whereas Y denoted for response, A, B, and C are the main affecting parameters, AB, AC and BC are interaction factors, A^2, B^2, and C^2 signifies quadratic effects of given factors, β_0 is constant arithmetic mean response, and β_1, β_2, β_3, β_{12}, β_{13}, β_{23}, β_{11}, β_{22}, β_{33} are estimated coefficients for the respected factors. The independent variables for optimization were albumin concentration (A), ethanol concentration (B), and agitation speed (C) at their low, medium and high level as represented in **Table S5**. Based on various statistical parameter software was evaluated and provided 3D response surface plot. The *p*-value evaluates the significance of factors based on the responses. Moreover, ANOVA was also applied to evaluate the significance of model and factors. The interactions between the selected process parameters were also evaluated by analyzing the contour plots and reach conclusive remarks.

Supplementary Table S5: Box-Behnken design: Factors and their levels

Process parameters	Unit	Levels		
		High	Medium	Low
Albumin	%w/v	6	4	2
Ethanol concentration	%v/v	100	70	40
Agitation speed	rpm	1000	750	500

Three levels for three main factors were selected in Box-Behnken design and based on it experiments were carried out on the basis of the runs obtained by the software.

Validation of QbD Design and Model. The optimized nanoplex was formulated by employing the experimental conditions as suggested in QbD Design **(Table S3).** The nanoplex was synthesized and evaluated based on the predicted and experimentally obtained responses. Further, the formulation was analyzed for its desired product attributes including targeted particle size and PDI. To ensure the desired product quality for evaluation of applied design space analyzed by validation of a model for the effect of every CPP on CQA.

Assessment of albumin purity. The purity of albumin used in the current investigation was checked via sodium dodecyl sulfate-polyacrylamide gel electrophoresis (SDS-PAGE), bicinchoninic acid assay (BCA assay) and MALDI-TOF/MS. The albumin was analyzed by SDS-PAGE for the presence of other components from the fraction V albumin. SDS-PAGE was carried out using stacking (5 %w/v) and resolving (10 %w/v) polyacrylamide gel. Albumin (15 µM) (n=6) was loaded on the gel and allowed to run (80 V for 1.5 hr). After electrophoresis, the gel was stained using Coomassie brilliant blue and followed by destaining with a solution containing 1:1:8::v:v:v; methanol: glacial acetic acid: water. The gel was seen under Chemiluminescent Gel Doc system (Bio-Rad Laboratories, California, USA)[14].

The BCA assay was performed to evaluate the concentration of albumin present in fraction V albumin. BCA assay was performed using the BCA protein assay kit (Pierce BCA Protein Assay Kit, Thermo Fischer Scientific, USA). Albumin (1 mg/ml) solution was prepared and mixed with the BCA working reagents (reagent A: B, 50:1). Here, protein sample preparation water was considered a blank solution. Protein samples were taken in a microplate well and incubated for 30 min at 37°C and absorbance was taken at 562 nm on a multimode plate reader (Varioskan LUX Multimode, Thermo Fisher Scientific, Massachusetts, USA). The concentration of albumin from nanoplex was calculated from the standard curve of albumin.

MALDI-TOF/MS was performed for proteomic analysis of the albumin. Using 5800 MALDI-TOF MS instrument (AB SCIEX, CA, USA). The albumin solution (1 mg/100 uL) was mixed with an equivalent amount of the matrix used Sinapinic Acid in 30 %v/v acetonitrile and 0.1 %v/v trifluoroacetic acid (sample:matrix= 1:5). A 1 µL sample was spotted on the target plate and evaporated under a mild stream of warm air. The mass spectra were developed in positive reflector mode with 25 kV accelerating voltage.

Confirmation of albumin presence in nanoplex. The presence of albumin in ANp, DTANp, siANp, and DTsiANp was confirmed via SDS-PAGE analysis as protocol mentioned above on stacking (5 %w/v) and resolving (10 %w/v) polyacrylamide gel. Equal concentration of albumin, ANp, DTANp, siANp, and DTsiANp (15 µM) was loaded on to the gel. Then, the gel was stained using Coomassie brilliant blue and followed by destained with a solution. The gel was visualized under Chemiluminescent Gel Doc system[14].

Albumin presence in nanoplex (ANp, DTANp, siANp, and DTsiANp) was further confirmed via evaluating albumin concentration from the ANp, DTANp, siANp, and DTsiANp with reference to albumin using BCA reagent assay kit. A 1mg/mL of each nanoplex and albumin were dissolved in DEPC treated RNase free water. The samples were mixed with BCA reagent A and B as mentioned above, then after incubating, absorbance was measured.

Confirmation of dendrimeric template presence in nanoplex. The incorporation of dendrimeric template in nanoplex was assessed via 2.3.3 2,4,6- Trinitrobenzene sulfonic acid (TNBSA) assay[15]. Briefly, ANp, siANp, DTANp and DTsiANp (5-100 µg/mL) were taken in 0.1 M sodium bicarbonate (pH 8.5) buffer. Then, the TNBSA (0.01 %w/v) solution was added and incubated at 37±0.5 °C for 2 h. Followed by 10 %w/v SDS and 1 N HCl was added to each sample to stop the reaction, and absorbance was measured at 335 nm in UV-visible spectrophotometer (Shimadzu, Kyoto, Japan) to determine the number of free amine groups. Number of albumin per gram was calculated with the help of the standard curve of glycine (2–20 µg/mL).

The incorporated dendrimeric template in nanoplex was further confirmed via surface charge evaluation. The ANp, siANp, DTANp and DTsiANp were diluted (10 times) using ultra-pure water and surface zeta potential was measured using Zetasizer (Nano-ZS90, Malvern Instruments, Worcestershire, UK).

RNase protection assay. To verify incorporation of siRNA inside the nanoplex and verify that the resultant architect is not the mere coprecipitate of siRNA and albumin, we performed the agarose gel electrophoresis experimentation. First of all, the prepared siANp and DTsiANp were reconstituted in DEPC treated RNase free water and centrifuged (21,000 g for 15 min at 4°C). The obtained pellet was collected was suspended in DEPC treated RNase free water and one portion of the pellet was treated with RNase enzyme (0.35µg/1 µg siRNA) for 30 min. It may be noted that the co-precipitate of siRNA or loosely attached siRNA were available to react with RNase

enzyme. In another control, the formed pellet was vigorously pipetted (10 min) and subsequently sonicated in an ultra sonicator for 30 min. This was done to mediate the breakage of nanoplex and release out the loaded siRNA from the nanoplex. Earlier prepared RNase treated pellet suspension and sonicated suspension of siANp and DTsiANp was loaded on agarose gel for evaluation of availability as well as stability of siRNA. On the other hand, as a reference, albumin siRNA (40mg: 200 pmol) and dendrimer-albumin-siRNA (40 mg albumin with selected *d*:siR ratio)) solution was incubated via stirring on a magnetic stirrer for 1 hr (1000 rpm) and siRNA was precipitated. The precipitated siRNA from albumin and albumin-dendrimer was also treated similarly with RNase enzyme (0.35µg/ 1 µg siRNA) for 30 min as mentioned for siANp and DTsiANp followed by loaded on the agarose gel.

Endo/lysosomal escape assay. The endo/lysosomal escape tendency of developed nanoplexes was performed on HG treated differentiated podocytes. Briefly, the podocytes were seeded on glass coverslips in 6 well culture plate ($1x10^6$ cells/well) and incubated for 24 h, then cells were treated with HG for 48 hr. After that, the cells were then treated with FAM-labeled siRNA (30 pmol; 3-4 µg/µL) loaded DTsiANp and siANp. After 6, 8 and 12 hr of incubation, podocytes were washed (3 times) with PBS (1X) and treated with Lysotracker dye (Thermo Fisher Scientific, Massachusetts, USA) for 30 min. Then, the podocytes cells were again washed with PBS (1X; 3 times) and fixed by Fluoroshield histology mounting medium (Sigma-Aldrich, Missouri, USA) on glass-slide (Borosil, Mumbai, India) and visualized using a Leica TCS SP5 AOBS Confocal microscopy system (Leica, Germany)[16].

Supplementary References

1 Fang, R., Shaozong, Y., Qian, H. & Wang, Y. (Google Patents, 2018).

2 Wu, L. *et al.* Albumin-based nanoparticles as methylprednisolone carriers for targeted delivery towards the neonatal Fc receptor in glomerular podocytes. *International journal of molecular medicine* **39**, 851-860 (2017).

3 Kamaly, N., He, J. C., Ausiello, D. A. & Farokhzad, O. C. Nanomedicines for renal disease: current status and future applications. *Nature Reviews Nephrology* **12**, 738 (2016).

4 Huang, J. *et al.* Quality by design case study: an integrated multivariate approach to drug product and process development. *International journal of pharmaceutics* **382**, 23-32 (2009).

5 Ban, E., Jang, D.-J., Kim, S.-J., Park, M. & Kim, A. Optimization of thermoreversible poloxamer gel system using QbD principle. *Pharmaceutical development and technology* **22**, 939-945 (2017).

6 Yerlikaya, F. *et al.* Development and evaluation of paclitaxel nanoparticles using a quality-by-design approach. *Journal of pharmaceutical sciences* **102**, 3748-3761 (2013).

7 Tiwari, D., Tiwari, R., Chandra, R., Bisen, P. & Haque, S. Efficient ELISA for diagnosis of active tuberculosis employing a cocktail of secretory proteins of Mycobacterium tuberculosis. *Folia biologica* **60**, 10 (2014).

8 Li, B. *et al.* MOFzyme: Intrinsic protease-like activity of Cu-MOF. *Scientific reports* **4**, 6759 (2014).

9 Cohn, E. J., Oncley, J. L., Strong, L. E., Hughes, W. L. & Armstrong, S. H. Chemical, clinical, and immunological studies on the products of human plasma fractionation. I. The characterization of the protein fractions of human plasma. *The Journal of clinical investigation* **23**, 417-432 (1944).

10 Kang, Y., Kim, H., Shin, W. S., Woo, G. & Moon, T. Effect of disulfide bond reduction on bovine serum albumin-stabilized emulsion gel formed by microbial transglutaminase. *Journal of food science* **68**, 2215-2220 (2003).

11 Luo, X., Tue, P.-T., Sugiyama, K. & Takamura, Y. High yield matrix-free ionization of biomolecules by pulse-heating ion source. *Scientific reports* **7**, 15170 (2017).

12 Tekade, R. K., Tekade, M., Kumar, M. & Chauhan, A. S. Dendrimer-stabilized smart-nanoparticle (DSSN) platform for targeted delivery of hydrophobic antitumor therapeutics. *Pharmaceutical research* **32**, 910-928 (2015).

13 Zhang, L. & Mao, S. Application of quality by design in the current drug development. *Asian journal of pharmaceutical sciences* **12**, 1-8 (2017).

14 Ono, M. *et al.* Radioiodination of BODIPY and its application to a nuclear and optical dual functional labeling agent for proteins and peptides. *Scientific reports* **7**, 3337 (2017).

15 Muniswamy, V. J. *et al.* 'Dendrimer-Cationized-Albumin'encrusted polymeric nanoparticle improves BBB penetration and anticancer activity of doxorubicin. *International journal of pharmaceutics* **555**, 77-99 (2019).

16 Han, J., Wang, Q., Zhang, Z., Gong, T. & Sun, X. Cationic bovine serum albumin based self-assembled nanoparticles as siRNA delivery vector for treating lung metastatic cancer. *Small* **10**, 524-535 (2014).
